# Pericyte phenotype switching alleviates immunosuppression and sensitizes vascularized tumors to immunotherapy in preclinical models

**DOI:** 10.1172/JCI179860

**Published:** 2024-09-17

**Authors:** Zhi-Jie Li, Bo He, Alice Domenichini, Jiulia Satiaputra, Kira H. Wood, Devina D. Lakhiani, Abate A. Bashaw, Lisa M. Nilsson, Ji Li, Edward R. Bastow, Anna Johansson-Percival, Elena Denisenko, Alistair R.R. Forrest, Suraj Sakaram, Rafael Carretero, Günter J. Hämmerling, Jonas A. Nilsson, Gabriel Y.F. Lee, Ruth Ganss

**Affiliations:** 1 Cancer Microenvironment Laboratory, Harry Perkins Institute of Medical Research, QEII Medical Centre and Centre for Medical Research, The University of Western Australia, Perth, Western Australia, Australia.; 2 Department of Geriatric Medicine, Shenzhen People’s Hospital (The Second Clinical Medical College, Jinan University, The First Affiliated Hospital, Southern University of Science and Technology), Shenzhen, Guangdong, China.; 3 Melanoma Discovery Laboratory, Harry Perkins Institute of Medical Research, QEII Medical Centre and Centre for Medical Research, The University of Western Australia, Perth, Western Australia, Australia.; 4 Sahlgrenska Center for Cancer Research, Department of Surgery, Institute of Clinical Sciences, University of Gothenburg and Sahlgrenska University Hospital, Gothenburg, Sweden.; 5 Systems Biology and Genomics Laboratory, Harry Perkins Institute of Medical Research, QEII Medical Centre and Centre for Medical Research, The University of Western Australia, Perth, Western Australia, Australia.; 6 INSiGENe Ltd., UGenome, Tucson, Arizona, USA.; 7 DKFZ–Bayer Immunotherapeutic Lab, German Cancer Research Center (DKFZ), Heidelberg, Germany.; 8 Tumorimmunology Program, DKFZ, Heidelberg, Germany.; 9 St. John of God Subiaco Hospital and School of Surgery, The University of Western Australia, Perth, Western Australia, Australia.

**Keywords:** Oncology, Therapeutics, Cancer immunotherapy, Mouse models, Pericytes

## Abstract

T cell–based immunotherapies are a promising therapeutic approach for multiple malignancies, but their efficacy is limited by tumor hypoxia arising from dysfunctional blood vessels. Here, we report that cell-intrinsic properties of a single vascular component, namely the pericyte, contribute to the control of tumor oxygenation, macrophage polarization, vessel inflammation, and T cell infiltration. Switching pericyte phenotype from a synthetic to a differentiated state reverses immune suppression and sensitizes tumors to adoptive T cell therapy, leading to regression of melanoma in mice. In melanoma patients, improved survival is correlated with enhanced pericyte maturity. Importantly, pericyte plasticity is regulated by signaling pathways converging on Rho kinase activity, with pericyte maturity being inducible by selective low-dose therapeutics that suppress pericyte MEK, AKT, or notch signaling. We also show that low-dose targeted anticancer therapy can durably change the tumor microenvironment without inducing adaptive resistance, creating a highly translatable pathway for redosing anticancer targeted therapies in combination with immunotherapy to improve outcome.

## Abstract 2

Pericyte plasticity in cancer is a new and so far unrecognize therapeutic vulnerability which can be targeted with low dose therapeutics to avoid adaptive resistance.

## Graphical abstract

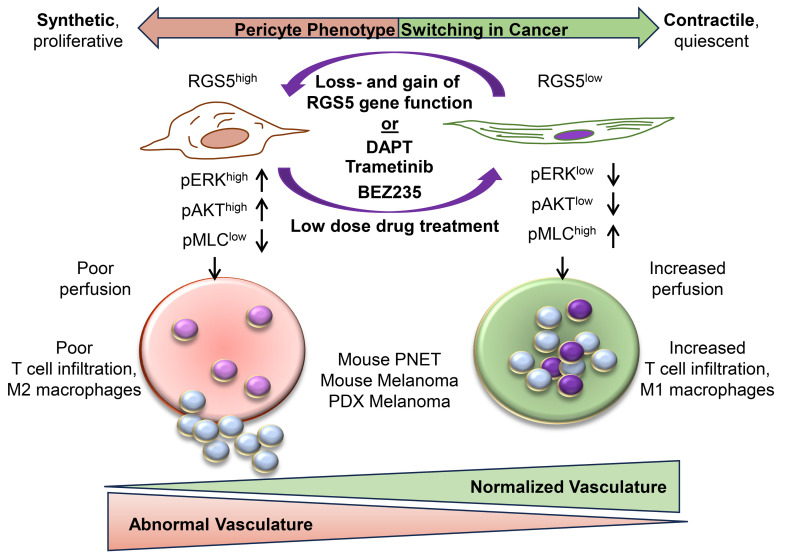

## Introduction

Tumor growth is driven by oncogenic signaling within a protumorigenic microenvironment. Cotargeting different tumor components has the potential to enhance anticancer treatments, including immunotherapies, but implementation of combination therapies is often hampered by lack of detailed mechanistic insights, toxicity, and adaptive resistance.

For instance, while targeted anticancer therapeutics using small molecule inhibitors or monoclonal antibodies interfering with pivotal oncogenic signaling have rapidly enhanced treatment for cancers such as melanoma, breast, and lung (1–3), clinical responses are often short-lived due to cancer cell heterogeneity and acquired resistance (4). Moreover, current treatment protocols employ maximal tolerated dose (MTD) ranges, which often cause considerable side effects. Ultimately, the clinical benefits of targeted therapies have been limited by lack of durability and marked toxicities. Given the greater recent success of immunotherapies using checkpoint inhibition or adoptive cellular therapy, ongoing clinical trials have combined these with targeted therapies to exploit mechanistically separate therapeutic arms (3). Even so, they can induce cross resistance; for instance, high-dose MAPK inhibition in melanoma can induce tumor immune evasion and failure of immune checkpoint blockade (5).

Given the limitations of combining immunotherapy with suppressing cancer cell signaling, an alternative is to combine immunotherapy with targeting the tumor stroma. For instance, combinations of immunotherapies with blockade of VEGF signaling are in clinical trials or have already been approved, e.g., for metastatic renal cell carcinoma (6, 7). Mechanistically, VEGF targeting in a dose-dependent manner promotes angiogenic vessel pruning or normalization (8) and can improve response to checkpoint inhibition (9–13). However, while VEGF depletion produces transient survival benefits in animal models, vessel normalization is short-lived and subsequent blood vessel regression increases tumor hypoxia, which in turn drives local invasion and metastases (14, 15). Thus, stroma-targeted therapeutic approaches are frequently unable to induce durable antitumor responses.

Since tumor vessel perfusion, not obliteration, strongly predicts responsiveness to immunotherapy (16), targeting vessel remodeling via pathways other than VEGF may open more durable opportunities for combination immunotherapies. The two major components of tumor blood vessels are endothelial cells and pericytes, which form the inner and outer layers of the vascular tube, respectively. Pericytes have long been overlooked in antiangiogenesis therapies, with only a few attempts at specific depletion by targeting PDGFR as an example (17, 18). Pericyte loss suppresses tumor growth temporarily, but similarly to what happens when targeting endothelial cells, increasing hypoxia subsequently fosters metastases (19, 20). Intratumoral pericytes are a heterogeneous population representing a spectrum of differentiation states (21). Regulator of G protein signaling 5 (RGS5), an intracellular regulator of G protein–coupled receptors, is prominently upregulated in the angiogenic vasculature in response to tumor hypoxia (22). Although RGS5 gene deletion normalizes the angiogenic vasculature (9, 22, 23), its role in regulating vascular phenotypes, specifically in relation to the pericyte, remains elusive. Mechanistic insights into these processes, for instance, resolving pericyte differentiation from elimination, are needed to create alternative opportunities to durably modulate tumor vasculature with wide-reaching applications for immune combination therapies.

## Results

### Cell-intrinsic properties are sufficient for regulating pericyte phenotype in vitro.

**Figure 1:**
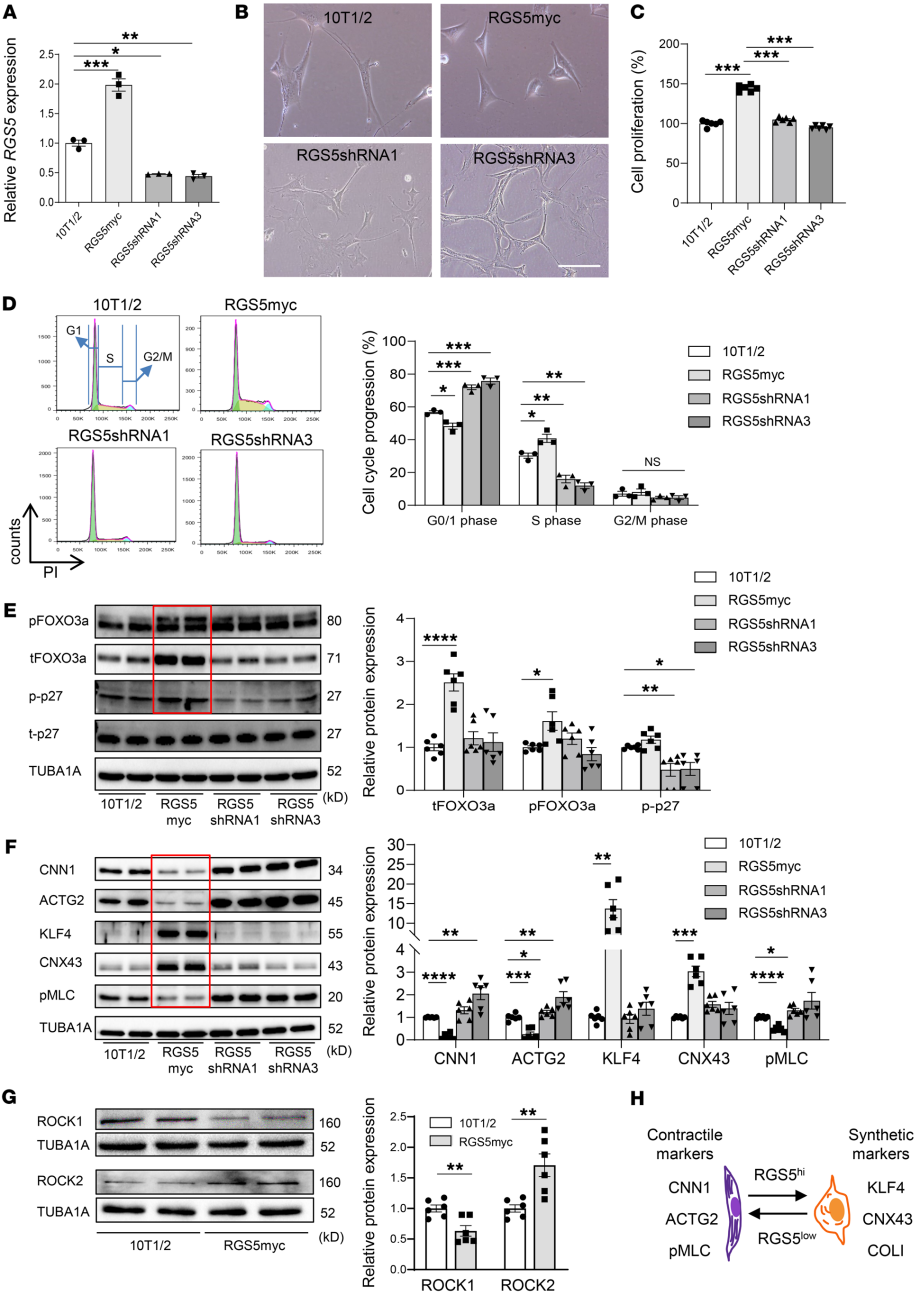
RGS5 expression levels regulate pericyte phenotype in vitro. (**A**) Relative *RGS5* mRNA expression in 10T1/2, RGS5myc overexpressing and *Rgs5* knockdown cell lines (RGS5shRNA1/3). *n* = 3 replica. Data are represented as mean ± SEM. *P < 0.01; **P < 0.001; ***P < 0.0001, 1-way ANOVA. (**B**) Microscopic images depicting 10T1/2, *RGS5* overexpressing, and *Rgs5* knockdown cells. Scale bar: 50 μm. (**C**) Cell proliferation (48 hours) in parental and transfectant 10T1/2 cells. *n* = 5 replica. Data are represented as mean ± SEM. ***P < 0.0001, 1-way ANOVA. (**D**) FACS blots showing PI histograms with color-coded cell cycle phases. Green, G0/G1 phase; yellow, S phase; blue, G2/M phase. Quantitative analysis of cell-cycle progression. *n* = 3 experiments. Data are represented as mean ± SEM. *P = 0.02; **P = 0.001; ***P = 0.0001, 1-way ANOVA. (**E**) Western blot (WB) of phosphorylated/total FOXO3a and phosphorylated/total p27KIP proteins. Red box highlights results in *RGS5* overexpression cells. Duplicates are shown for each marker. Relative phosphorylated and total FOXO3a expression were normalized to tubulin; relative phosphorylated p27KIP was normalized to total p27KIP expression and quantified. *n* = 3 experiments (2 replica each). Data are represented as mean ± SEM. tFOXOa: ****P < 0.0001; pFOXO3a: *P = 0.028; p-p27: *P = 0.020, **P = 0.006, 1-way ANOVA. (**F**) WB of contractile (CNN1, ACTG2) and synthetic (KLF4, CNX43) markers in correlation to Rho kinase activity (p-MLC). Red box highlights results in *RGS5* overexpression cells. Duplicates are shown for each marker, and relative protein expression normalized to tubulin was quantified. *n* = 3 experiments (2 replica each). Data are represented as mean ± SEM. CNN1: ****P < 0.0001, **P = 0.011; ACTG2: ***P = 0.0001, **P = 0.012, *P = 0.047; KLF4: **P = 0.003; CNX43: ***P = 0.003; p-MLC: ****P < 0.0001, *P = 0.017, 1-way ANOVA. (**G**) WB of ROCK1 and ROCK2 proteins. Duplicates are shown for each marker. Relative protein expression normalized to tubulin was quantified. *n* = 3 experiments (2 replica each). Data are represented as mean ± SEM. **P = 0.005, Student’s *t* test. (**H**) Contractile and synthetic pericyte markers in relation to RGS5 high (Rgs5^hi^) or low (*Rgs5*^low^) expression.

Pericytes play an integral role during tumor angiogenesis and together with endothelial cells control vessel integrity. Importantly, their capacity to switch between proliferative and quiescent states might be harnessed to improve vascular function in cancer (24). We have previously shown that *RGS5* is highly expressed in the tumor vasculature, most likely in pericytes (9). However, *RGS5* expression in primary pericytes is lost upon culture (21), thus requiring generation of an in vitro model to analyze the intrinsic role of RGS5 in pericyte maturation. 10T1/2 cells are not pericytes, but this model was chosen because, similar to pericytes, cells are of mesenchymal origin and can differentiate into a smooth muscle cell–like (SMC-like) phenotype, evidenced by induction of SMC-specific genes such as calponin (*Cnn1*), caldesmon (*Cald1*), alpha 2 smooth muscle actin (*Acta2*), and gamma 2 actin (*Actg2*) (24, 25). Native 10T1/2 cells were stably transfected with a myc-tagged RGS5 construct (RGS5myc), resulting in 10T1/2 RGS5myc cells showing a 2-fold increase in *RGS5* mRNA. In contrast, *Rgs5* gene knockdown using lentiviral shRNA (RGS5shRNA1 and -3) reduced endogenous *RGS5* expression levels to 50% of that of native 10T1/2 cells (Figure 1A). High *RGS5* expression was accompanied by morphological cell changes from spindle-shaped into a polygonal phenotype (Figure 1B). *RGS5* overexpressing cell lines proliferated more strongly than 10T1/2 or *RGS5* knockdown cells (Figure 1C) and underwent a cell-cycle shift from G0/G1 to S and G2/M phases (Figure 1D). This phenotype correlated with an increase in total and phosphorylated forkhead transcription factor 3a (FOXO3a), which promotes cell survival (26), and enhanced phosphorylation of p27KIP, which increases cell mobility and cell-cycle progression (27) (Figure 1E). Importantly, increased *RGS5* expression reduced expression of contractile markers (CNN1, ACTG2) and increased vascular synthetic markers such as Krüppel-like factor 4 (KLF4) and connexin 43 (CNX43) (28, 29) compared with WT 10T1/2 cells. Conversely, knocking down *RGS5* stimulated differentiation of parental 10T1/2 cells, as evidenced by increase of CNN1/ACTG2 and reduction of KLF4/CNX43 (Figure 1F). Enhanced contractile marker expression strongly correlated with increased expression of phosphorylated myosin light chain (p-MLC), a surrogate marker for Rho kinase (Rho-associated protein kinase [ROCK]) activity (Figure 1F). Furthermore, in angiogenic pericytes (10T1/2 RGS5myc cells) Rho kinase isoform 1 (ROCK 1) is downregulated, whereas ROCK 2 is upregulated, suggesting distinct roles of these isoforms in regulating pericyte maturity (Figure 1G). Overall, these results imply that RGS5 levels can be altered to affect the maturation levels of pericytes in vitro, and quiescent and more mature pericytes also show enhanced Rho kinase activity (Figure 1H).

### Pericyte phenotype regulates tumor-vessel normalization and abnormalization.

**Figure 2:**
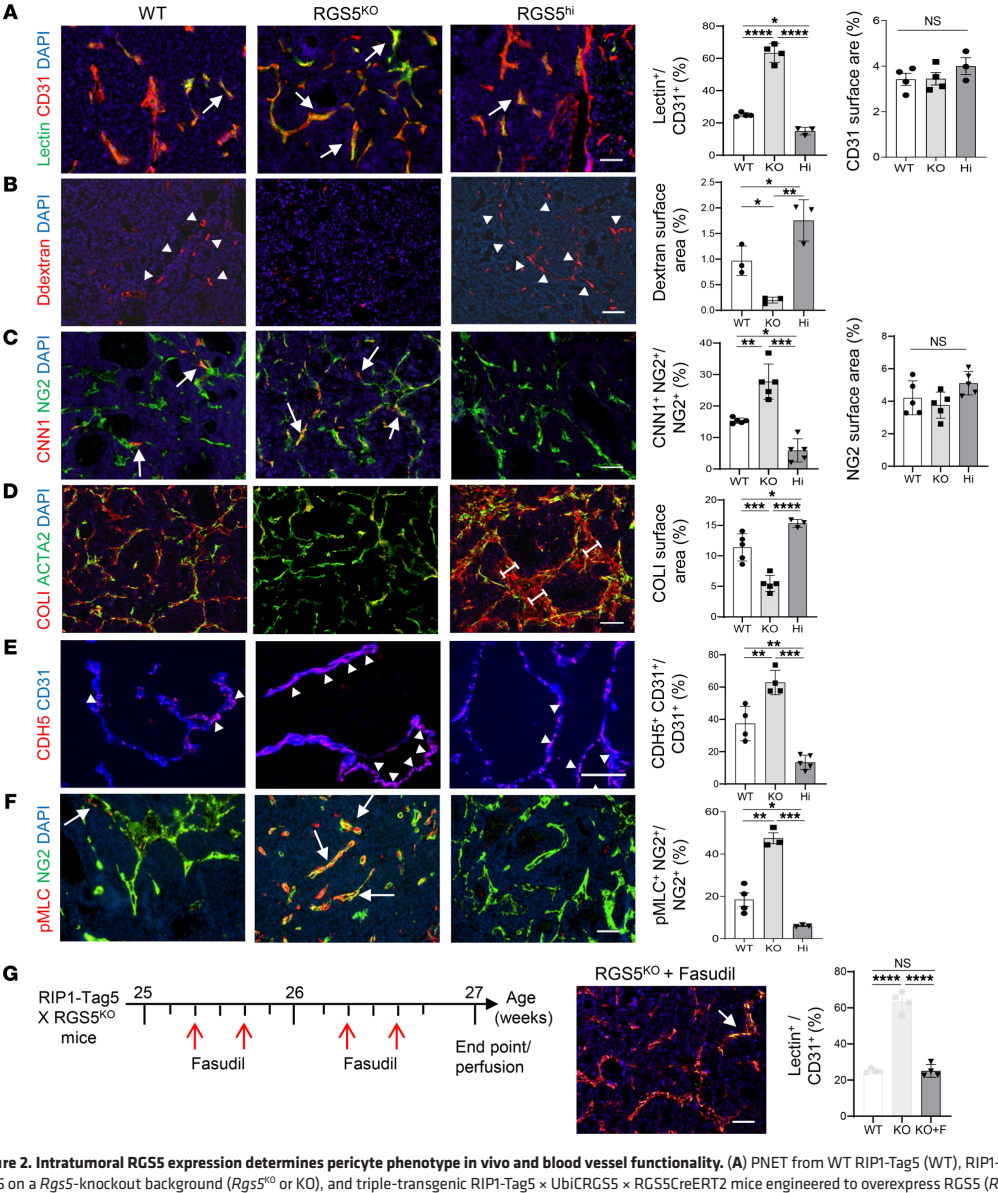
Intratumoral RGS5 expression determines pericyte phenotype in vivo and blood vessel functionality. (**A**) PNET from WT RIP1-Tag5 (WT), RIP1-Tag5 on a *Rgs5*-knockout background (*Rgs5*^KO^ or KO), and triple-transgenic RIP1-Tag5 × UbiCRGS5 × RGS5CreERT2 mice engineered to overexpress RGS5 (*Rgs5*^hi^ or HI) were analyzed at 27 weeks. Images depict vascular CD31 expression (red) and infused FITC-lectin (green) as surrogate markers for tumor perfusion; arrows indicate overlay (yellow). Quantification of overlay and CD31 vessel area. *n* = 3–4 mice. *P = 0.02; ****P < 0.0001, 1-way ANOVA. (**B**) Extravasation of 70 kD dextran (red, arrowheads) from blood vessels into tumor parenchyma as marker for vessel leakiness. *n* = 3 mice. Data are represented as mean ± SEM. *P = 0.04; **P = 0.0014, 1-way ANOVA. (**C**) Calponin (CNN1, red) expression in pericytes (NG2, green); arrows indicate overlay (yellow); quantification of overlay and frequency of NG2^+^ pericytes. *n* = 5 mice, *P = 0.0064; **P = 0.0008; ***P < 0.0001, 1-way ANOVA. (**D**) COLI (red) deposition around pericytes (ACTA2, green); brackets indicate width of COLI deposits. *n* = 3–5 mice. Data are represented as mean ± SEM. *P = 0.022; ***P = 0.0006; ****P < 0.0001, 1-way ANOVA. (**E**) VE-cadherin (CDH5, red, arrowheads) coverage of CD31 (blue) vessels, *n* = 4–5 mice. Data are represented as mean ± SEM. **P = 0.0023; ***P < 0.0001, 1-way ANOVA. (**F**) p-MLC (red) expression in pericytes (NG2, green); arrows indicate overlay (yellow), *n* = 3–4 mice. Data are represented as mean ± SEM. *P = 0.03; **P = 0.0003; ***P < 0.0001, 1-way ANOVA. (**G**) Fasudil treatment schematic of *Rgs5*^KO^ PNET mice and assessment of tumor perfusion at endpoint. CD31 (red) overlay with infused FITC-lectin (yellow) is highlighted by arrows. Perfusion was quantified in *Rgs5*^KO^+fasudil (**F**) group in comparison with WT and *Rgs5*^KO^ (data from **A**, shadowed). *n* = 4–5 mice. Data are represented as mean ± SEM. ****P < 0.0001, 1-way ANOVA. Scale bars: 50 μm (**A**–**D**, **F**, **G**); 25 μm (**E**).

While tumor blood vessels have been therapeutic targets for decades, it is still poorly understood how vessel abnormalities could be durably reversed. Single-cell RNA-Seq (scRNA-Seq) of tumors univocally demonstrated that *RGS5* is highly and exclusively upregulated in tumor pericytes (Supplemental Figure 1A; supplemental material available online with this article; https://doi.org/10.1172/JCI179860DS1) (9, 23). This provides a unique opportunity for studying the role of pericyte phenotype switching during angiogenic blood-vessel remodeling in vivo. To this end, *Rgs5* loss- (*Rgs5*^KO^) (9) and gain-of-function (*Rgs5*^hi^) pancreatic neuroendocrine tumors (PNET, RIP1-Tag5) were established (Supplemental Figure 1, B and C). These tumors develop in a background of global RGS5 gene deletion/overexpression. Functionally, reduced tumor perfusion and vessel leakiness, both hallmarks of an angiogenic tumor vasculature, were evident in WT PNET tumors (Figure 2, A and B). In contrast, in *Rgs5*^KO^ tumors, perfusion increased 3-fold, as evidenced by enhanced FITC-lectin staining (Figure 2A), which coincided with vascular leakiness for high molecular dextran reducing by 4-fold (Figure 2B). In *Rgs5*^hi^ tumors, perfusion was significantly reduced (*P* = 0.02) and vascular leakiness was enhanced (*P* = 0.04) when compared with WT RIP1-Tag5 tumors (Figure 2, A and B). Moreover, vessel normalization in *Rgs5*^KO^ mice was associated with increased expression of the contractile marker CNN1 in tumor pericytes, whereas vascular expression of the synthetic marker collagen I (COLI) was reduced. Highly aberrant vessels in *Rgs5*^hi^ tumors displayed a pericyte phenotype opposite of that of *Rgs5*^KO^ tumors, namely low CNN1 expression and large vascular COLI deposits indicative of excessive extracellular matrix (ECM) production by synthetic pericytes (28) (Figure 2, C and D). Phenotype switching of pericytes also affected endothelial barrier function, as demonstrated by a more continuous and increased vascular endothelial–cadherin (VE-cadherin) expression pattern along blood vessels in *Rgs5*^KO^ compared with irregular and sparse VE-cadherin expression in WT and *Rgs5*^hi^ tumors (Figure 2E). Similar to what was seen in 10T1/2 in vitro data, the synthetic pericyte state in vivo correlated with low p-MLC expression, whereas upregulation of vascular p-MLC coincided with expression of contractile markers (Figure 2F). Indeed, Rho kinase activity is crucial to vessel normalization, since treatment of *Rgs5*^KO^ PNET-bearing mice with the Rho kinase inhibitor fasudil reduced tumor perfusion to WT levels (Figure 2G). A similar relationship between pericyte phenotype switching and vascular p-MLC expression was observed in OVA-expressing B16 melanoma (B16-OVA) (30), with upregulation of contractile markers and p-MLC in *Rgs5*^KO^ compared with WT C57BL/6 tumors (Supplemental Figure 2, A–C). Overall, these data demonstrate that the intrinsic RGS5 levels in pericytes control angiogenic vessel remodeling and pericyte differentiation in a process dependent on pericyte-specific Rho kinase activity.

### Pericyte phenotype switching is sufficient for creating an immune-supportive tumor microenvironment.

**Figure 3:**
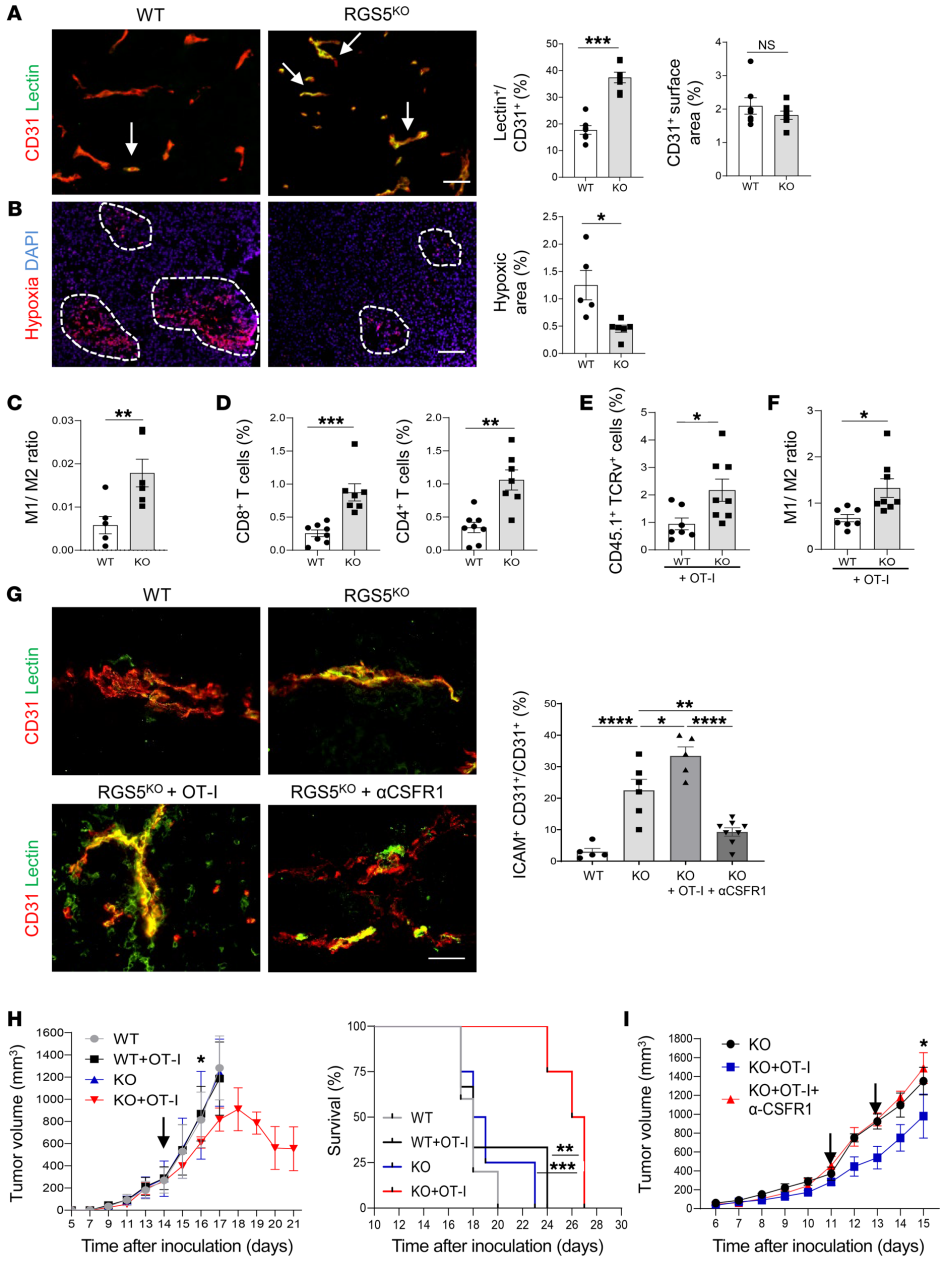
“Forced” pericyte maturation changes the tumor microenvironment and enhances immunotherapy. (**A**) B16-OVA tumors in WT or R*gs5*^KO^ (KO) mice. Overlay (yellow, arrows) of FITC-lectin (green) with CD31^+^ vessels. *n* = 5 mice. Data are represented as mean ± SEM. ***P < 0.0001, Student’s *t* test. (**B**) Hypoxy probe accumulation (red circles). *n* = 5–6 mice. Data are represented as mean ± SEM. *P = 0.01, Student’s *t* test. (**C**) M1/M2 macrophage ratio. *n* = 4–6 mice. Data are represented as mean ± SEM. **P = 0.01, Student’s *t* test. (**D**) Endogenous T cells. *n* = 7–8 mice. Data are represented as mean ± SEM. **P = 0.008; ***P = 0.0004, Student’s *t* test. (**E**) OT-I CD8^+^ T cells (CD45.1^+^TCRv2α^+^). *n* = 7–8 mice. Data are represented as mean ± SEM. *P = 0.024, Student’s *t* test. (**F**) Macrophage M1/ M2 ratio after OT-I transfer. *n* = 7–8 mice. Data are represented as mean ± SEM. *P = 0.014, Student’s *t* test. (**G**) ICAM (green) expression on vessels (red). *n* = 5–8 mice. Data are represented as mean ± SEM. *P = 0.013; **P = 0.0013; ****P < 0.0001, 1-way ANOVA. (**H**) B16-OVA mice, untreated or treated with adoptive transfer (arrow). Tumor growth, *n* = 4–6 mice. Data are represented as mean ± SEM. *P = 0.019 on day 16, WT+OT-I versus *Rgs5*^KO^+OT-I, multiple unpaired *t* tests. **P = 0.012, *Rgs5*^KO^ versus *Rgs5*^KO^+OT-I; ***P = 0.0067, WT+OT-I versus *Rgs5*^KO^+OT-I, log-rank (Mantel-Cox) test. (**I**) *Rgs5*^KO^ mice, untreated or treated with adoptive OT-I cell transfers (arrows) or transfers combined with αCSF1R. *n* = 3–4 mice. Data are represented as mean ± SEM. *P = 0.013 *Rgs5*^KO^+OT-I versus *Rgs5*^KO^+OT-I+anti-CSF1R on day 15, multiple unpaired *t* tests. Scale bars: 50 μm (**A**); 100 μm (**B**); 25 μm (**G**).

Intratumoral hypoxia drives cancer progression and immune escape. A high hypoxia score indicates poor prognosis and treatment resistance in multiple cancer types, including melanoma (31). It is, however, still unclear how angiogenesis and tumor oxygenation control the tumor immune environment. Pericyte phenotype switching in B16-OVA tumors increased lectin perfusion in *Rgs5*^KO^ hosts compared with WT mice and also strongly reduced hypoxia in the tumor microenvironment (Figure 3, A and B). To determine whether the change in the pericyte physiological state in *Rgs5*^KO^ B16-OVA tumors could reshape the immune environment, in particular, macrophage polarization status, intratumoral macrophages were analyzed by FACS for the expression of M1- or M2-like markers (32). An increase in M1-like macrophages was found in *Rgs5*^KO^ B16-OVA tumors, while M2-like macrophage numbers remained unchanged when compared with WT B16-OVA (Figure 3C and Supplemental Figure 3, A and B). Intratumoral macrophages in *Rgs5*^KO^ tumors expressed fewer angiogenic factors such as *VEGFA* and *MMP9* (Supplemental Figure 3C). The shift in macrophage polarization in *Rgs5*^KO^ B16-OVA tumors also correlated with increased numbers of endogenous CD4^+^ and CD8^+^ T cells in the tumor environment (Figure 3D and Supplemental Figure 3D). Similarly, preactivated, adoptively transferred congenic H-2K^b^/OVA-specific TCR transgenic T cells (OT-I) (33) homed more efficiently into *Rgs5*^KO^ compared with WT B16-OVA tumors (*P* = 0.024) (Figure 3E and Supplemental Figure 3E). Following adoptive T cell transfer, M1 macrophages were increased in both WT and *Rgs5*^KO^ tumors when compared with tumors without OT-I transfer, but the ratio of M1/M2-like macrophages remained elevated on a *Rgs5*^KO^ background compared with WT (Figure 3F and Supplemental Figure 3F). Furthermore, ICAM-1 expression, a marker for activated endothelium, strongly correlated with the frequency of M1-like macrophages and T cells in the tumor microenvironment. Conversely, macrophage depletion in *Rgs5*^KO^ mice with anti-CSF receptor (αCSF1R) antibody treatment reduced ICAM expression in tumor vessels (Figure 3G), demonstrating the biological relevance of macrophage polarization in vivo. Treatment with a single OT-I adoptive transfer decreased tumor burden and significantly increased survival of *Rgs5*-deficient B16-OVA–bearing mice when compared with mice with WT background (*P* = 0.0124, Figure 3H). The responsiveness of *Rgs5*^KO^ B16-OVA tumors to OT-I transfer was abolished with anti-CSFR antibody treatment, which deleted 82% of intratumoral macrophages (Figure 3I and Supplemental Figure 3G). Thus, the genetic switch to a more mature pericyte phenotype substantially contributes to a cascade of events that includes reduction of hypoxia, M1 macrophage polarization, and blood vessel inflammation, which opens the tumor for antitumor effector T cells and subsequent immune-mediated tumor destruction.

### Drug-induced pericyte maturation mimics loss of RGS5.

**Figure 4:**
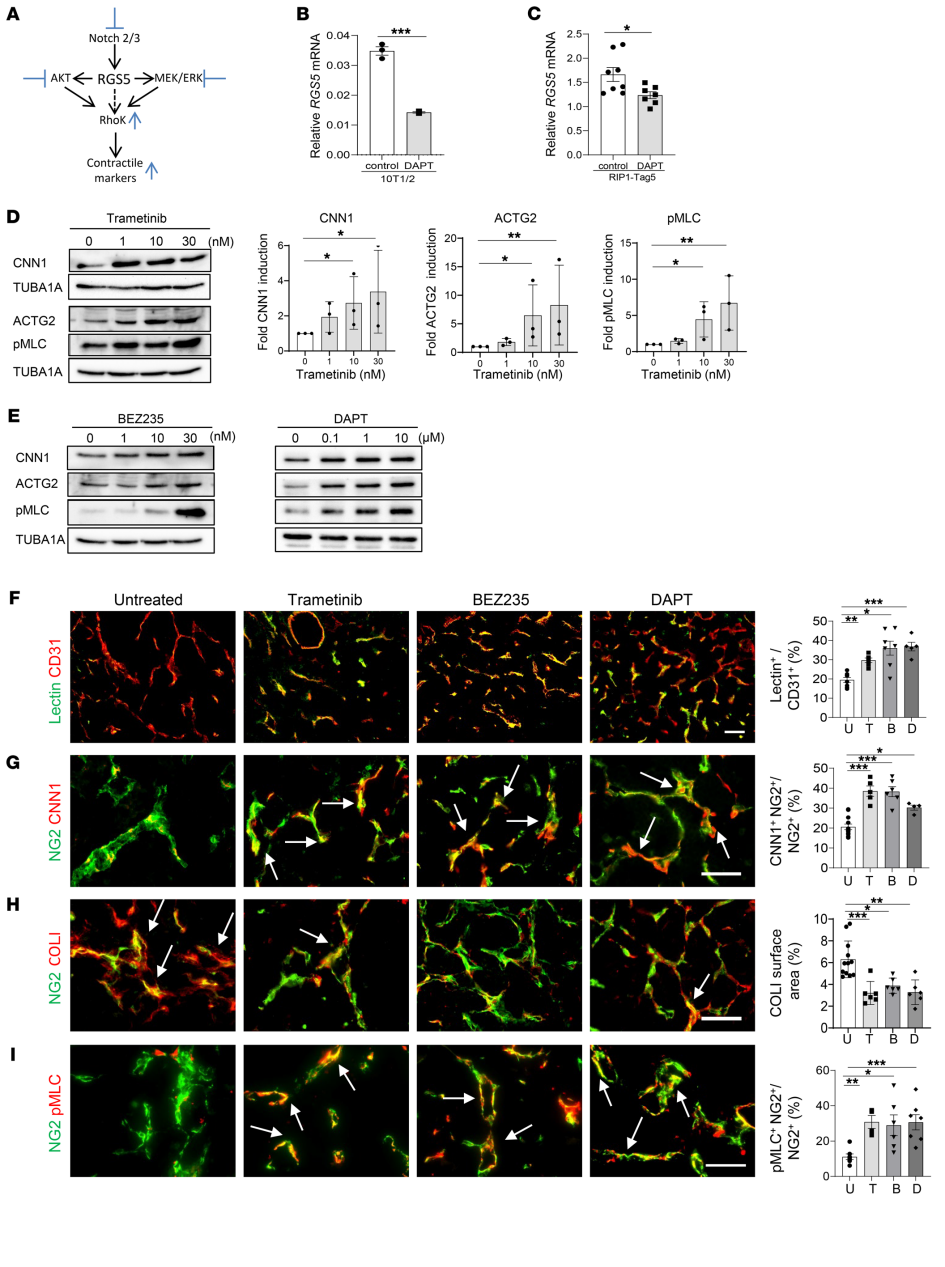
Low-dose therapeutics mimic *Rgs5* knockdown by inducing pericyte maturation. (**A**) RGS5 signaling and Rho kinase–activating effects of inhibitors (blue bars). (**B**) Relative *RGS5* expression in 10T1/2 cells, 40 μM DAPT. *n* = 3 biological replica. Data are represented as mean ± SEM. ***P = 0.0001, Student’s *t* test. (**C**) Relative *RGS5* expression in RIP1-Tag5 tumors treated with DAPT. *n* = 7–8 mice. Data are represented as mean ± SEM. *P = 0.025, Student’s *t* test. (**D**) Contractile markers (CNN1, ACTG2) and p-MLC in RGS5myc cells with increasing doses of trametinib. Quantification of 3 independent experiments. Data are represented as mean ± SEM. *P ≤ 0.04, **P ≤ 0.006, 1-way ANOVA (Kruskal-Wallis test). (**E**) Representative WB of CNN1, ACTG2, and p-MLC in RGS5myc cells with increasing doses of BEZ235 (left) and DAPT (right). The experiment was conducted twice. (**F**) RIP1-Tag5 mice untreated (U) or treated with trametinib (T), BEZ235 (B), or DAPT (D). FITC-lectin overlay (yellow) with CD31^+^ (red) vessels was quantified. *n* = 4–12. Data are represented as mean ± SEM. *P = 0.0173; **P = 0.0001; ***P < 0.0001, 1-way ANOVA. (**G**) Quantification of CNN1 expression (red) in relation to NG2^+^ pericytes (green). Arrows indicate overlay (yellow), *n* = 6–8. Data are represented as mean ± SEM. *P = 0.022, ***P < 0.0001, 1-way ANOVA. (**H**) COLI deposition (red) around NG2^+^ pericytes (green). Arrows indicate overlay of markers (yellow). *n* = 6–12. Data are represented as mean ± SEM. *P = 0.006; **P = 0.0006; ***P = 0.0005, 1-way ANOVA. (**I**) p-MLC expression in NG2^+^ pericytes (green). Arrows indicate overlay (yellow). *n* = 4–7. Data are represented as mean ± SEM. *P = 0.016; **P = 0.014; ***P = 0.0053, 1-way ANOVA. Scale bars: 100 μm (**F**); 50 μm (**G**–**I**).

Having demonstrated in genetic models that “forced” pericyte maturation contributes to effective immunotherapy, we next investigated potential pharmacological targets related to RGS5 signaling (Figure 4A). RGS5 regulates major pathways such as AKT/PI3K and MEK/ERK (22), which are also linked to Rho kinase activity and vascular SMC (vSMC) contractility (34, 35). Indeed, in tumor pericytes, phospho-S6 ribosomal protein (p-S6R, a downstream target of AKT signaling) and p-ERK were downregulated in normalized *Rgs5*^KO^, but upregulated in highly abnormal *Rgs5*^hi^ tumor vessels compared with WT PNET (Supplemental Figure 4). Furthermore, RGS5 itself is a potential target of notch signaling (36) and *RGS5* mRNA expression can be suppressed by treatment of pericytes (10T1/2) in vitro or PNET-bearing mice in vivo with the notch inhibitor DAPT (*N*-[*N*-(3,5-diflouorophenylacetyl-l-alanyl)]-S-phenylglycerinet-butyl ester, γ secretase inhibitor, GSI-IX) (37) (Figure 4, B and C). To analyze the effects of targeted therapy on pericyte maturation in vitro, the MEK inhibitor trametinib (GSK1120212) (38), the dual PI3K/mTOR inhibitor BEZ235 (Dactolisib, Novartis; ref. 39), and DAPT were selected to treat angiogenic pericytes (10T1/2mycRGS5myc). Blockade of MEK, AKT, or notch signaling in pericytes in vitro restored contractile marker expression in correlation with upregulation of p-MLC (Figure 4, D and E). Induction of pericyte differentiation is pathway specific, since other inhibitors such as AG490 (JAK/STAT inhibitor) were ineffective (Supplemental Figure 5A). Contractile marker induction is also dose dependent and is suppressed with high-dose drug treatment, as shown for BEZ235 (Supplemental Figure 5B). Trametinib, BEZ235, and DAPT at MTD show broad anticancer activities in multiple cancer models by inducing cell-cycle arrest, apoptosis, and growth inhibition (37–39). Importantly, for this subsequent part of the study, we aimed for low-dose drug applications to induce pericyte differentiation without reducing tumor burden or decreasing tumor blood-vessel numbers. To this end, PNET-bearing mice were treated with 0.02 mg/kg trametinib, 10 mg/kg BEZ235, or 5 mg/kg DAPT for a total of 10 days. These treatments did not reduce pericyte or endothelial cell numbers or cancer cell proliferation as measured by Ki67 staining; they did, however, downregulate vascular ERK (p-ERK) and AKT (p-S6R) signaling (Supplemental Figure 5, C and D). Importantly, with low-dose drug treatment, a normalized vasculature was induced, which resulted in improved tumor perfusion compared with untreated tumors (Figure 4F). Indeed, the vessel normalization was accompanied by increased CNN1 expression in pericytes (Figure 4G), reduced vascular COLI deposits (Figure 4H), and enhanced vascular p-MLC signals (Figure 4I), demonstrating that low-dose inhibition of MEK, AKT, or notch signaling pathways induced pericyte phenotype switching in vivo. In addition, in drug-treated PNET tumors, similar to R*gs5*^KO^ tumors, blood-vessel diameters were reduced and pericytes were more closely aligned with endothelial cells compared with untreated tumors (Supplemental Figure 6, A–C), consistent with vascular stabilization. These data provide proof of concept that strategic low-dose pharmacological intervention can normalize angiogenic vessels and mimic the vascular changes induced by genetic deletion of the *Rgs5* gene.

### Low-dose targeted therapy alleviates immunosuppression in the tumor microenvironment.

**Figure 5:**
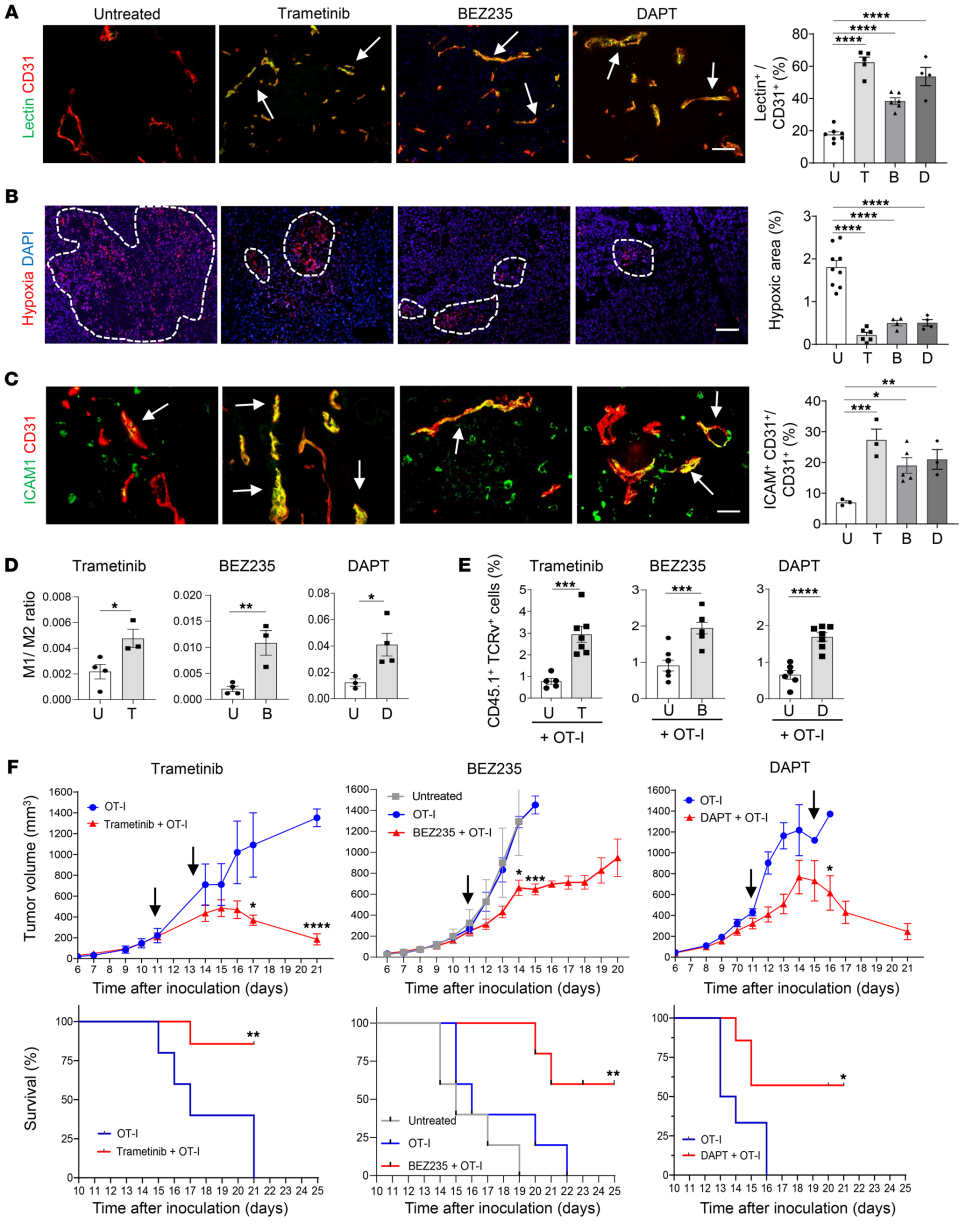
Low-dose therapeutics improve effectiveness of anticancer immunotherapy. (**A**) B16-OVA tumors untreated or treated with trametinib, BEZ235, or DAPT. Quantification of FITC-lectin (green) overlay (yellow, arrows) with CD31^+^ (red) blood vessels. *n* = 4–7 mice. Data are represented as mean ± SEM. ****P < 0.0001, 1-way ANOVA. (**B**) Quantification of hypoxy probe (red, circles). *n* = 4–9 mice. Data are represented as mean ± SEM. ****P < 0.0001, 1-way ANOVA. (**C**) Quantification of vascular (CD31^+^, red) ICAM (green) expression (yellow, arrows). *n* = 3–5 mice. Data are represented as mean ± SEM. *P = 0.042; **P = 0.034; ***P = 0.0033, 1-way ANOVA. (**D**) Tumors untreated or treated with trametinib, BEZ235, or DAPT. M1/M2 macrophage ratio. *n* = 3–4 tumors. Data are represented as mean ± SEM. *P = 0.034; **P = 0.008; *P = 0.0272 (DAPT), Student’s *t* test. (**E**) Quantification of OT-I T cells (CD45.1^+^TCRv2α^+^), following adoptive transfer, groups as in **D**. *n* = 5–7 mice. Data are represented as mean ± SEM. ***P = 0.004; ****P ≤ 0.0001, Student’s *t* test. (**F**) B16-OVA mice untreated or treated with drugs before OT-I cell transfers (arrows). Trametinib: *n* = 5–7, mean ± SEM. Tumor growth on days 17 and 21, *P = 0.012; ****P < 0.0001, multiple unpaired *t* tests. Survival: **P = 0.0039, log-rank (Mantel-Cox) test. BEZ235: *n* = 5 mice, mean ± SEM. Tumor growth on days 13 and 14. *P = 0.014; ***P < 0.0001, multiple unpaired *t* tests. Survival: **P = 0.0039 OT-I versus BEZ235+OT-I, log-rank (Mantel-Cox) test. DAPT: *n* = 5 mice, mean ± SEM. Tumor growth on day 16. *P = 0.04, multiple unpaired *t* tests. Survival: *P = 0.0276, log-rank (Mantel-Cox) test. Scale bars: 100 μm (**A**, **B**); 25 μm (**C**).

To further assess the potential benefits of drug-induced vessel normalization, we analyzed secondary changes in the tumor microenvironment and implications for immunotherapy. C57BL/6 mice were implanted with B16-OVA tumors, and once tumors were palpable, mice were treated with low-dose trametinib, BEZ235, or DAPT daily. These low-dose treatments alone had no impact on B16-OVA tumor growth (Supplemental Figure 7A). Importantly, however, perfusion of B16-OVA tumors was increased in correlation with reduced tumor hypoxia; vascular ICAM expression was increased, reminiscent of B16-OVA tumors implanted in *Rgs5*^KO^ mice (Figure 5, A–C). Next, we treated B16-OVA tumors with 6 daily drug applications, followed by analysis of macrophage phenotypes. In treated B16-OVA tumors, the frequency of M1-like macrophages was increased, resulting in elevated M1/M2 macrophage ratios when compared with untreated tumors (Figure 5D and Supplemental Figure 7, B–D). A 2-fold increase in OT-I T cells was detected after transfer into drug-treated B16-OVA tumors compared with control tumors, also leading to a dramatic increase in macrophage infiltration, with a 2-fold increase in the M1/M2 ratio (Figure 5E and Supplemental Figure 7E). Survival studies then demonstrated delayed tumor outgrowth and increased survival for all low-dose drug treatment groups compared with what occurred in untreated B16-OVA tumors or OT-I transfers alone (Figure 5F). To further consolidate the role of the vasculature as a major target for low-dose drug treatment, tumors harboring an already normalized vasculature (*Rgs5*^KO^) were treated with low-dose trametinib and adoptive T cell therapy. As shown in Supplemental Figure 8A, effector T cells reduced tumor growth in the absence of the *Rgs5* gene (see also Figure 3H) with no additive trametinib effects. Tumors in a genetic *Rgs5*^hi^ background, harboring more abnormal blood vessels than WT tumors, were less responsive to treatment with low-dose trametinib and T cell therapy compared with tumors in WT mice. Similarly, high-dose trametinib (1 mg/kg) treatment delayed tumor growth per se, but reduced responsiveness to OT-I transfers (Supplemental Figure 8B). High trametinib doses (1 and 2 mg/kg) caused cancer cell death, tumor necrosis (Supplemental Figure 8, C and D), loss of blood vessels, and pericyte coverage of remaining vessels (Supplemental Figure 8, E and F), demonstrating the crucial role of vessel status for immunotherapy. Only low-dose drug treatment acted synergistically with adoptive T cell transfers in correlation with improved tumor perfusion (Supplemental Figure 8, B and E). Thus, pharmacological induction of pericyte maturation/vascular normalization by low-dose drug treatment profoundly ameliorates hypoxia, facilitates immune cell entry into the tumor microenvironment, and renders adoptive T cell therapy more effective.

### Drug-induced pericyte phenotype switching is highly durable.

**Figure 6:**
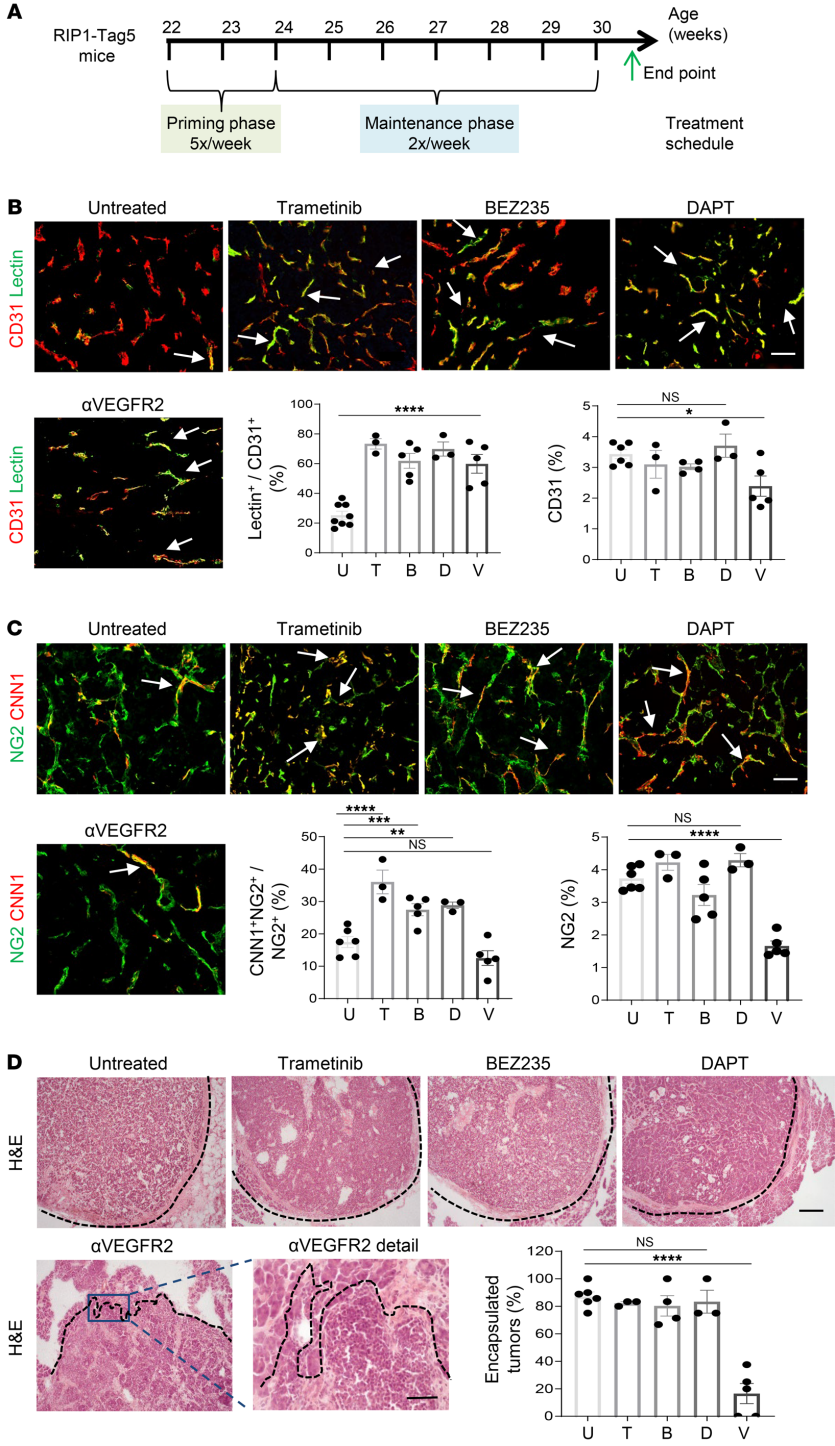
Tumor-vessel normalization following long-term treatment with low-dose therapeutics is highly sustainable. (**A**) Eight-week treatment schematic in RIP1-Tag5 mice including 2-week priming and 6-week maintenance phase. (**B**) Representative images of untreated mice or mice treated with trametinib (0.02 mg/kg), BEZ235 (5 mg/kg), DAPT (5 mg/kg), or anti-VEGFR2 antibodies (V, DC101, 15 mg/kg). FITC-lectin (green) overlay (yellow, arrows) with CD31^+^ (red) blood vessels was quantified as surrogate marker for tumor perfusion. *n* = 3–8 mice. Data are represented as mean ± SEM. ****P < 0.0001 for all treatment groups compared with untreated. Quantification of CD31^+^ intratumoral blood vessels, *P = 0.031, NS, not statistically significant for trametinib, BEZ235, and DAPT treatments compared with untreated, 1-way ANOVA. Scale bar: 100 μm. (**C**) Representative images and quantification of overlay (yellow, arrows) of CNN1 (red) expressing NG2^+^ pericytes (green). *n* = 3–6 mice. Data are represented as mean ± SEM. **P = 0.009; ***P = 0.007; ****P < 0.0001. Quantification of NG2^+^ intratumoral pericytes: ****P < 0.0001, NS, not statistically significant for trametinib, BEZ235, and DAPT treatments compared with untreated, 1-way ANOVA. Scale bar: 50 μm. (**D**) Representative H&E images and quantification of percentage of RIP1-Tag5 tumors displaying an intact collagen capsule (dotted line). *n* = 3–6 mice. Data are represented as mean ± SEM. ****P < 0.0001, NS, not statistically significant for trametinib, BEZ235, and DAPT treatments compared with untreated, 1-way ANOVA. Scale bars: 200 μm (upper images and αVEGFR2); 50 μm (αVEGFR2 detail).

Antiangiogenic agents, in particular anti-VEGF/R therapy at low dose, can change the immunosuppressive microenvironment to enhance immunotherapy (40). However, chronic treatment outcomes may differ from short-term results observed in implantation tumor models. To differentiate between long-term vascular remodeling via pericyte maturation and low-dose anti-VEGFR2 targeting, PNET-bearing mice at 22 weeks of age were treated with 15 mg/kg DC101 (low-dose anti-VEGFR2 antibody application) (40), trametinib, BEZ235, or DAPT over an extended period of 8 weeks (Figure 6A). Tumors at endpoint in all treatment groups showed 2- to 3-fold increased perfusion when compared with untreated PNET, consistent with the notion that vascular normalization can be effected by targeting tumor pericytes or endothelium (8). However, even low-dose DC101 treatment reduced overall vascularity, indicating antiangiogenic effects and vessel loss with chronic inhibition of VEGF signaling. In contrast, trametinib, BEZ235, and DAPT did not affect CD31^+^ vessel numbers in comparison with what occurred in WT tumors (Figure 6B). Further, pericyte contractile markers such as CNN1 remained upregulated with no loss of pericyte coverage of endothelial cells following long-term trametinib, BEZ235, or DAPT treatments compared with WT tumors; DC101-treated tumors did not change pericyte phenotype, but reduced overall pericyte numbers (Figure 6C). Importantly, low-dose DC101 treatment caused breakdown of the collagen-rich tumor capsule, likely as a consequence of hypoxic pressure, which is a first step in local tumor invasion and propensity for metastatic dissemination in PNET (14). In contrast, trametinib, BEZ235, or DAPT treatments had no effects on tumor encapsulation (Figure 6D). These data show that vessel normalization following “forced” pericyte maturation is maintained during extended treatment periods. Ultimately, maintaining a functioning vasculature and an immune-supportive environment provides longer term opportunities for combination immunotherapies.

### Pericyte phenotype is a therapeutic vulnerability in human cancers that creates opportunities for targeted therapy.

**Figure 7:**
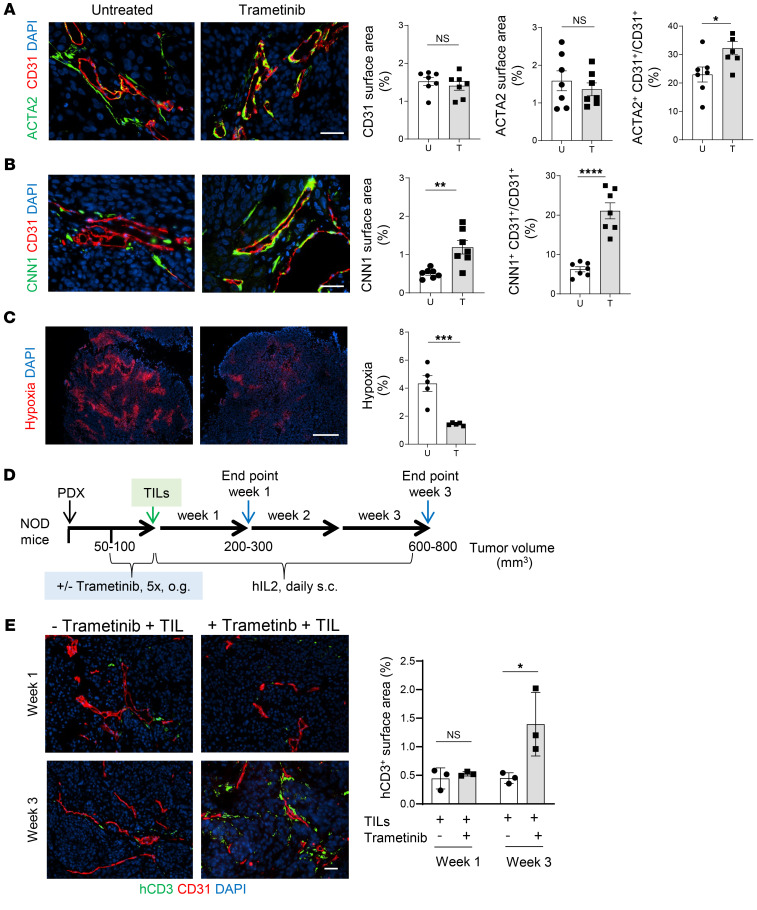
Pericyte phenotype switching is active in melanoma PDX and facilitates TIL tumor uptake. (**A**) NSG mice were implanted with melanoma PDX and treated with 10 doses of trametinib (0.02 mg/kg, o.g.) or left untreated. Representative images of blood vessels (CD31^+^) and pericytes (ACTA2^+^) in untreated and trametinib-treated PDX melanoma tumors. Quantification of total CD31^+^ vessels (red), total ACTA2^+^ (green) pericytes, and ACTA2^+^ covered CD31^+^ blood vessels (yellow). *n* = 7 mice. Data are represented as mean ± SEM. *P = 0.0224, Student’s *t* test. Scale bar: 50 μm. (**B**) Representative images and quantification of total CNN1 (green) expression and CNN1^+^ (green) covered CD31^+^ blood vessels (red). *n* = 7 mice. Data are represented as mean ± SEM. **P = 0.0026; ****P < 0.0001, Student’s *t* test. Scale bar: 50 μm. (**C**) Quantification of tumor hypoxia in treatment groups (red hypoxy probe deposits). *n* = 5 mice. Data are represented as mean ± SEM. ***P = 0.0009, Student’s *t* test. Scale bar: 500 μm. (**D**) Treatment schematic of melanoma PDX tumor–bearing NSG mice with autologous TILs and time line for analysis. (**E**) Representative images depicting human CD3^+^ TIL infiltration (green) at weeks 1 or 3 into melanoma PDX left untreated or pretreated with 5 doses of trametinib before TIL transfer. Quantification of infiltrating TILs 1 week and 3 weeks after adoptive transfer into PDX mice. *n* = 3 mice. Data are represented as mean ± SEM. *P = 0.01, 1-way ANOVA. Scale bar: 50 μm.

Trametinib is clinically approved for the treatment of NRAS/BRAF-mutated metastatic melanoma (41); however, data showing efficacy in combination with immunotherapies are still lacking (42). To assess the effects of low-dose trametinib treatment in an in vivo model of human cancer, a patient-derived xenograft (PDX) model carrying melanoma was employed. PDX mice were treated with 10 doses of trametinib followed by analysis of tumor vascular status and oxygenation. While CD31^+^ blood vessel numbers and overall ACTA2^+^ pericyte coverage were not affected by the treatment, pericytes aligned more closely to the vasculature following trametinib treatment and expression of the pericyte contractile marker CNN1 was significantly (*P* ≤ 0.0001) increased (Figure 7, A and B). Indeed, low-dose MEK inhibition significantly reduced tumor hypoxia, most likely due to vessel normalization (*P* = 0.0009, Figure 7C). Moreover, consistent with vessel-normalization effects, pretreatment of melanoma PDX-bearing mice with trametinib before transfer of ex vivo–expanded autologous tumor-infiltrating lymphocytes (TILs) resulted in significantly improved TIL penetration into tumors over time (*P* = 0.01, Figure 7, D and E).

**Figure 8:**
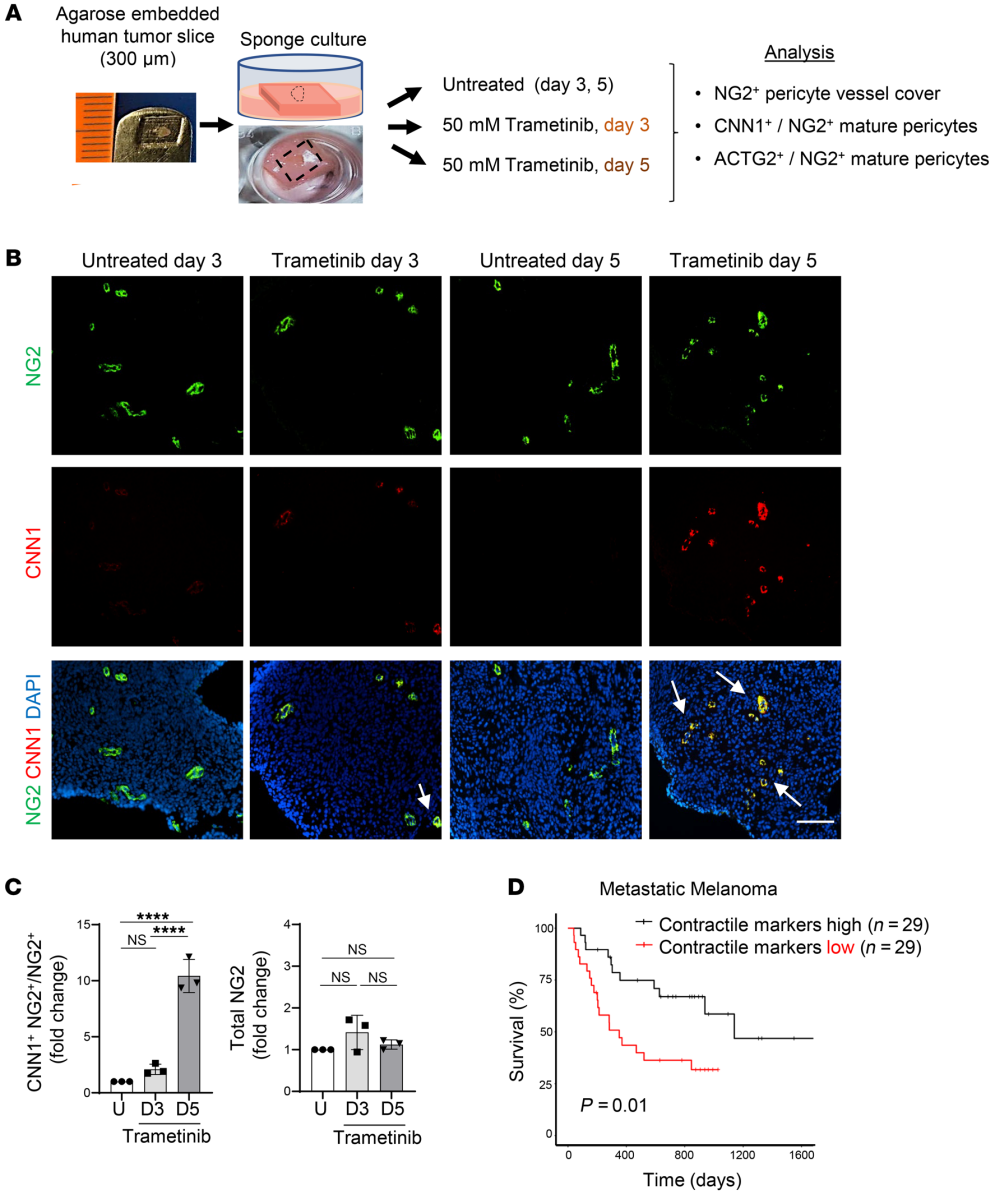
Pericyte phenotype switching is inducible in human cancer and correlates with melanoma patient survival. (**A)** Schematic of ex vivo organ slice culture; 1 to 2 mm^2^ diameter/300 μm thick human meningioma sections in agarose were cultured on sponge material in media in 24-well plates, and vascular markers were analyzed by immunohistochemistry after 3 and 5 days in culture with or without trametinib. (**B**) Microscopic images of meningioma tumor slices cultured for 3 or 5 days with or without trametinib. CNN1 staining (red) depicts mature CNN1^+^ covered (yellow, arrows) and NG2^+^ (green) pericytes. Scale bar: 100 μm. (**C**) Quantification of total NG2 signals and CNN1 covered NG2^+^ pericytes in untreated meningioma slices (day 3, day 5) and slices incubated with 50 mM trametinib for 3 and 5 days (D3, D5). *n* = 3 patients. Data are represented as mean ± SEM. ****P < 0.0001, 1-way ANOVA. (**D**) Prognostic value of a contractile gene signature for disease progression in a metastatic melanoma patient cohort comparing top and bottom expression quartiles (*n* = 29 patients each). *P* = 0.01, log-rank test.

To provide evidence that pericyte maturation can be induced in human tumor blood vessels, an ex vivo organ slice culture of primary intracranial neoplasms arising from the membranous layer of the central nervous system (meningiomas) was employed. Tumor sections of 300 μm were left untreated or treated with 50 nM trametinib for 3 and 5 days in culture (Figure 8A). Pericyte numbers were maintained during the culture period. In addition, MEK inhibition induced expression of the contractile markers CNN1 and ACTG2 in NG2^+^ pericytes over time (Figure 8, B and C, and Supplemental Figure 9). This finding provides proof of concept that pericyte maturation can be induced in human blood vessels by using a clinically approved drug at a dosing substantially below its MTD. To assess whether expression of contractile markers may affect patient survival, a contractile gene signature was generated and gene expression data from a cohort of metastatic melanoma patients were analyzed (43). High expression of a contractile gene signature indeed positively correlated with overall patient survival (*P* = 0.01, Figure 8D). In summary, these data underscore the importance of pericyte phenotype in cancer survival and treatment outcome and as therapeutic targets.

## Discussion

This study demonstrates that angiogenic pericytes can be “forced” to switch from a highly proliferative to a more mature phenotype by targeting intrinsic pathways that lead to activation of Rho kinase. Rho kinase activity in turn regulates the shape, proliferation, and movement of pericytes by acting on the cytoskeleton. This is an exciting biological concept that explores natural pericyte physiology in cancer and provides markers to assess vessel normalization in relation to patient outcomes as well as unexplored drug applications.

Our studies into RGS5-related signaling in pericytes uncovered that proliferating *Rgs5*^hi^ pericytes phenotypically manifest a less differentiated state that can be reversed by reducing RGS5 levels to induce procontractile signaling, which in turn improves overall vessel stability. Importantly, alterations in the pericyte maturation state do not affect their viability, thus enabling durable effects on vascular remodeling. In fact, pericyte maturation as shown here is reminiscent of vSMC phenotype switching, which is an integral part of wound healing (28), but disturbed in cancer (44).

Pericyte contractile markers in cancer remain largely unexplored. We have previously reported that extrinsic factors such as TGF-β can induce pericyte contractile marker expression in the tumor vascular niche, consistent with TGF-β being a differentiation factor for mural precursors such as 10T1/2 cells (24, 25). Furthermore, low-dose photodynamic therapy targeted to the vasculature induced pericyte but not endothelial cell contractile properties, resulting in improved vascular function (45). A common underlying theme of contractile marker upregulation in tumor pericytes, whether intrinsically or extrinsically controlled, seems to be Rho kinase activation, suggesting that pericyte Rho GTP signaling and its downstream target ROCK provide essential cues for microvascular stabilization (24, 45, 46). Considering the 2 ROCK isoforms, ROCK1-mediated p-MLC activation leads to pericyte differentiation, which stabilizes vessels, whereas ROCK2 signaling in tumor pericytes seems to impair vascular function (47). These findings underscore the vital role of pericyte maturation in regulating the entire vascular bed. Previous studies have linked vessel normalization with improved immunotherapy, but normalizing agents often affect both endothelial cells and immune cells, making it difficult to separate these effects (12, 13, 40). Collectively, RGS5 gain- and loss-of-function studies demonstrate the compelling link between pericyte differentiation, vascular normalization and activation, and enhanced efficacy of immunotherapies. The focus of this study was on intratumoral effects; however, systemic effects in a global RGS5 knockout or following systemic drug treatments cannot be excluded.

Most importantly, our data also provide a strong rationale for combining low-dose targeted therapy to induce pericyte differentiation with immunotherapy. In the absence of specific RGS5 small-molecule inhibitors (48), this study focused on targeting RGS5 signaling pathways, leading to upregulation of Rho kinase activity as well as contractile proteins. MEK/ERK and PI3K/AKT/mTOR signaling pathways are likely to regulate primal cellular responses, including survival, proliferation, and migration, in all intratumoral cells in a dose-dependent manner. However, this study focused on low-dose MEK/ERK and PI3K/AKT/mTOR pathway inhibition, which induced pericyte differentiation and overall vascular remodeling, but not cancer cell–growth inhibition (49, 50). Critically, pericyte targeting has durable vascular effects, unlike antiangiogenic VEGF blockade, when assessed in a transgenic tumor model over an 8-week treatment course, a substantially longer time frame than usual for animal models (14, 15). Importantly, we also showed that the concept of reinstalling expression of pericyte contractile markers with low-dose drug treatment is applicable for human cancer, and indeed a high expression score of contractile markers positively correlates with melanoma patient outcomes.

MEK inhibitors were initially developed to target oncogenic signaling in cancer cells, but have also shown immune stimulatory properties. However, clinical trials combining MEK inhibitors with immunotherapy have so far yielded conflicting results (5, 42). While MEK depletion in cancer cells increased tumor immunogenicity and enhanced T cell infiltration, systemic high-dose MEK inhibition impaired T cell activation, which could be reversed by engagement of costimulatory receptors such as 4-1BB, CD40, and OX40 (51–55). Of note, in these preclinical studies, the orally active MEK inhibitor trametinib was applied at doses several hundred–fold higher than in our low-dose study. We also show that high-dose drug treatment fails to act synergistically with immunotherapy, most likely because vascular dysfunctions inherent to tumor growth are not improved.

Similar to MEK inhibition, but clinically less developed, is the concept of targeting PI3K/AKT in combination with immunotherapy (56). Beneficial effects with checkpoint inhibitors were observed in preclinical models of castration-resistant prostate cancer in which AKT inhibition specifically suppressed myeloid cells, but preserved T cell function (57). Interestingly, in human xenograft and transgenic mammary cancer models, RAS-PI3K-AKT inhibitors at a dose that did not reduce tumor growth increased tumor perfusion and reduced hypoxia (58). However, none of these studies provided a mechanistic link between vessel normalization and T cell infiltration. Nevertheless, these findings support our rationale of repurposing low-dose drug approaches that increase pericyte contractility and reduce hypoxia for combination therapies with the potential of durable effects.

Notch inhibition with γ-secretase inhibitors has so far failed to demonstrate clinical benefits in most solid cancers (59). Investigations into immune combination therapies are scarce, most likely because notch signaling is essential for CD8^+^ T cell effector function and notch inhibition may promote regulatory T cell–mediated immune suppression (60, 61). However, in a mouse model of pancreatic xenografts, a notch 2/3–specific antibody (OMP-59R5, tarextumab) downregulated stromal RGS5 mRNA expression concomitant with vascular maturation, supporting our RGS5–notch 3 link (36). Our findings suggest the need to revisit notch inhibition in anticancer therapy by dose modulation and in combination with immune effectors.

Overall, extensive preclinical and clinical studies demonstrate that high-dose targeting of oncogenic signaling pathways in cancer cells is short-lived due to selective pressure and that loss of stromal components increases microenvironmental stress and resistance. Combination with immunotherapy might be promising, but high-dose targeted therapy is cytotoxic and poses a real risk of negatively affecting antitumor immunity over time (5, 42). Recent alternative strategies for targeted therapies support the concept of inducing physiological and durable changes rather than widespread toxicity. For example, simultaneous low-dose application of RAF, MEK, and ERK inhibitors decreased the selective pressure of each compound and overcame acquired EGFR resistance in lung cancer (62). Combination of MEK and CDK4/6 inhibitors in KRAS-positive pancreatic cancer induced cancer cell senescence and created a physiological stress response that activated endothelial cells and sensitized tumors to PD-1 checkpoint blockade (63).

While we focused here on low-dose targeting of MEK, PI3K/AKT, and notch signaling for vessel normalization, presumably other therapeutics can also reduce pericyte proliferation in situ. This study introduces mechanistic insights that allow the exploration of targeted therapy at doses that do not interfere with overactivated oncogenic signaling or impair cancer growth per se. Instead, exploiting the phenotypic plasticity of pericytes affords highly durable vascular changes and “preconditions” the microenvironment, which then improves subsequent immune therapies. Furthermore, by modifying the dosing and timing of clinically approved drugs, our study immediately addresses the urgent need for drugs that specifically and durably modulate the tumor microenvironment and act synergistically with emerging immunotherapies.

## Methods

### Sex as a biological variable.

Our study examined male and female animals that were randomly assigned to experimental groups as well as male and female human specimens, and similar findings are reported for both sexes.

### Cell lines.

Murine C3H10T1/2 (10T1/2) cells were purchased from ATCC. RGS5-specific small hairpins (RGS5shRNA1 and 3) were generated using the following sequences: RGS5shRNA1, top: GATCCGCTATGGATTTGCCAGCTTCATTCAAGAGATGAAGCTGGCAAATCCATAGCTTTTTTACGCGTG, bottom: AATTCACGCGTAAAAAAGCTATGGATTTGCCAGCTTCATCTCTTGAATGAAGCTGGCAAATCCATAGCG; and RGS5shRNA3, top: GATCCGCGGAGAAGGCAAAGCAAATTTTCAAGAGAAATTTGCTTTGCCTTCTCCGCTTTTTTACGCGTG, bottom: AATTCACGCGTAAAAAAGCGGAGAAGGCAAAGCAAATTTCTCTTGAAAATTTGCTTTGCCTTCTCCGCG. These were then cloned into the lentiviral expression vector pLVX-shRNA2 (Clontech), which coexpresses the fluorescent protein ZsGreen1. Myc-tagged RGS5 (RGS5myc) was generated using the following primers: forward: ATTACTCGAGATGTGTAAGGGACTGGCAGCTCTG; reverse: GCCGGATCCTTACAGATCCTCTTCTGAGATGAGTTTTTGTTCCTTGATTAGCTCCTTATAAAATTC. RGS5myc was then cloned into the lentiviral expression vector pLVX-IRES-ZsGreen1 (Clontech). Lentiviral particles were generated using a HEK 293/17–based packaging system (ATCC). 10T1/2 parental cells were infected with lentiviruses and enriched for high GFP expression by FACS. B16-OVA cells are C57BL/6 murine B16 melanoma cells transfected with OVA (MO4) (30).

### Cell-proliferation assay.

To measure cell proliferation of 10T1/2 RGS5 transfectants, 2 × 10^3^ cells/well were seeded in complete DMEM medium in a 96-well plate and cultured for 24 or 48 hours. Culture medium was replaced with fresh complete medium containing MTS solution (CellTiter 96 AQueous One Solution Cell Proliferation Assay, Promega) at a ratio of 20 μl MTS/100 μl medium. The plate was incubated for 3 hours in a humidified incubator at 5% CO_2_. The absorbance at 490 nm was recorded in a plate reader (LUMIstar Omega, BMG Labtech), and cell proliferation was analyzed with GraphPad Prism software (version 10.1.2).

### Mouse models.

All mice were kept in pathogen-free facilities at The University of Western Australia or the Harry Perkins Institute, with food and water provided ad libitum. RIP1-Tag5 mice express SV40 large T antigen under the control of the rat insulin promoter and were bred on a C3HeBFe (C3H) background as previously described (64). *Rgs5*-knockout mice (*Rgs5*^KO^) were generated by crossing RGS5LoxP mice (LoxP flanked first exon of the *Rgs5* gene) with CreDeleter mice as published (9) and backcrossed on a C57BL/6 (C57BL/6JOzarc, Ozgene Pty. Ltd.) background. *RGS5*-overexpressing mice on a C57BL/6 background (RGS5^hi^) were generated by intercrossing RGS5CreERT2 (inducible Cre recombinase knockin into RGS5 locus, exon 2) and UbiCRGS5 (ubiquitin-driven RGS5 gene knockin into Rosa 26 locus) mice (see also Supplemental Figure 1B; both strains generated by Ozgene). PDX tumors were implanted into nonobese diabetic severe combined immunodeficiency *IL2rg-*knockout mice (NOD.Cg-Prkdc^scid^Il2rg^tm1Wjl^/SzJ, NSG, Ozgene).

### Human specimen.

Fresh human meningioma specimens were collected at the time of surgical resection, kept on ice in culture medium, and prepared for organ slice culture within 30 to 40 minutes after resection. Five specimens were collected from 4 female and 1 male patient. For the melanoma PDX model, a biopsy from a patient with in-transit metastatic melanoma was collected.

### Immunotherapy.

For tumor studies, RIP1-Tag5 mice were used (WT) or crossed with *Rgs5*-deficient mice (*Rgs5*^KO^) or UbiCRGS5 × RGS5CreERT2 double-transgenic mice treated with 5 daily injections of 20 mg/kg 4-hydroxytamoxifen (tamoxifen, Cayman) in corn oil (MilliporeSigma) at 24 to 25 weeks of age (*Rgs5*^hi^). Tumors were analyzed at 27 to 28 weeks. For melanoma induction, 1 × 10^6^ B16-OVA cells were injected intradermally into the flanks or abdomens of mice (32). Congenic (CD45.1) H-2K^b^–restricted, OVA-specific TCR transgenic T cells (OT-I) (33) were activated in vitro with 10 U/ml IL-2 (PeproTech) and 25 nM OVA peptide 257–264 (SIINFEKL, GenScript) for 3 days. For adoptive transfers, 0.5 to 2 × 10^6^ activated OT-I T cells were injected i.v. into mice at indicated time points. For macrophage depletion, mice were injected with 250 μg antimouse CSF1R antibodies (Bio X Cell) at days 9, 10, and 12 following tumor cell inoculation. At end stage, mice were anesthetized followed by transcardiac perfusion with 2% formalin. Prior to sacrifice, some mice were i.v. injected with 50 μg FITC-labeled tomato lectin (*Lycopersicon esculentum*, Vector, circulated for 10 minutes), pimonidazole (60 mg/kg, circulated for 60 minutes, Hypoxyprobe-1 Kit, Hypoxyprobe Inc.), or 1 mg of 70 kD TRITC-labeled dextran (Invitrogen, 10 minutes circulation). B16-OVA tumor burden was assessed by measurement of length and width with a microcaliper and calculated using the following formula: (length × width^2^)/2. The ethical end point was reached when tumors measured 1,500 mm^3^.

### Melanoma PDX tumor model.

The melanoma PDX model was generated via serial transplantation of a patient biopsy into immunocompromised mice. Briefly, tumor tissue from patients or mice was dispersed into single cells, mixed 1:1 with Matrigel, and injected s.c. into the flanks of NSG mice. When tumors reached 500–1,500 mm^3^, they were serially transplanted into new recipient mice. PDX mice carrying tumors of 50–150 mm^3^ were treated with vehicle or 0.02 mg/kg trametinib by oral gavage (o.g.). Mice were treated 5 times per week for 2 weeks and sacrificed on day 3 after the last treatment. Tumors were fixed in formalin for histological analyses. To assess immune-cell infiltration, PDX mice carrying tumors of 50–100 mm^3^ were treated with vehicle or 0.02 mg/kg trametinib by o.g. for 5 consecutive days, followed by i.v. injection of 1 × 10^7^ ex vivo–expanded autologous TILs. TILs were generated using a rapid expansion protocol as previously published (65). Briefly, patient-derived tumor pieces were cultured in high-dose human recombinant IL-2 media (PeproTech) for isolation of TILs. TILs were rapidly expanded in the presence of IL-2, anti-human CD3 antibodies (Miltenyi Biotec), and irradiated human PBMCs. Tumors were frozen in OCT medium and analyzed for the presence of human T cells by immunohistochemistry.

### In vivo drug treatment.

The following drugs, dosing, and application routes were used for in vivo treatments: BEZ235 (dactolisib, NSP-BEZ235, Selleck Chemicals, 5 or 10 mg/kg, o.g., in 10% MMP [methyl 3-([2,2-dimethylbutanoyl]thio)propanoate]/90% PEG 300), DAPT (GSI-IX, Selleck Chemicals, 5 or 10 mg/kg, o.g. in 5% DMSO/corn oil), fasudil hydrochloride (fasudil, LKT Laboratories, 30 mg/kg, i.p. 0.9% saline), trametinib (GSK1120212, Selleck Chemicals, 0.006 or 0.02 mg/kg [low dose], 1 and 2 mg/kg [high dose], o.g., in 5% DMSO and 95% PEG300). For VEGF-blocking studies, 15 mg/kg anti-VEGFR2 antibodies (DC101, Bio X Cell, in PBS) were injected i.p.

### Western blot analysis.

10T1/2 RGS5 transfectants were incubated in complete DMEM medium or serum starved for 24 hours followed by stimulation. Cells were washed twice with PBS and lysed in RIPA buffer containing PMSF and protease and phosphatase inhibitor cocktails (MilliporeSigma). Protein concentration was quantified using the bicinchoninic acid (BCA) Assay Kit (Thermo Fisher). A total of 20 μg protein was separated on a 12% SDS-PAGE gel and transferred onto a PVDF membrane (MilliporeSigma). The membrane was incubated with blocking buffer (20 mM Tris, 150 mM NaCl, 0.1% Tween 20, 5% skim milk powder, pH 7.6) for 1 hour at room temperature, followed by incubation with primary antibodies in blocking buffer overnight at 4°C (primary antibodies, Supplemental Table 1). Membranes were incubated with HRP-conjugated secondary antibodies (secondary antibodies, Supplemental Table 2) and protein expression visualized with enhanced chemiluminescence solution (Thermo Fisher Scientific), and quantified using the ChemiDoc MP Imaging System (Bio-Rad, version 6.1).

### IHC.

Mice were perfused with 2% formalin, and tumors were isolated, post-fixed in formalin, and paraffin embedded or incubated in 10% sucrose (2 hours) followed by 30% sucrose overnight and frozen in OCT compound (Tissue Tek). Ice-cold acetone was used to fix 7 μm frozen sections before IHC. Paraffin sections were deparaffinized, rehydrated, and quenched in 3% H_2_O_2_ in H_2_O, followed by antigen retrieval. Primary antibodies are listed in Supplemental Table 1. Primary antibodies were detected using the M.O.M. (Mouse on Mouse) Immunodetection Kit, fluorescein (Vector), streptavidin-conjugated (SA-conjugated) Alexa Fluor 594 (Thermo Fisher Scientific), and fluorescent-conjugated anti-IgG antibodies (secondary antibodies, Supplemental Table 2). DAPI was used in some tissues to visualize cell nuclei and quantify necrosis. Hypoxia was quantified in mice treated with pimonidazole using anti-pimonidazole antibodies (Hypoxyprobe Inc.). A Nikon Ti-E microscope and NIS software (Nikon, version 4.0) were used for image analysis. At least 3 mice or tumors were analyzed per treatment group; 5–15 images per tumor were analyzed. All material summarized in 1 graph was imaged with standardized threshold intensity. Positively stained features are expressed as percentage of marker expression compared with total tumor surface area in 1 image (surface area percentage). Vessel diameters were determined by dividing vessel area by its length (24), and 30–40 vessels/mouse were analyzed. Alternatively, colocalization was measured as fluorescence intensity ratio between red and green fluorescence channels or percentage overlay of red/green fluorescence.

### Flow cytometry.

To investigate cell-cycle progression in 10T1/2 RGS5 transfectants, cells were cultured in 10 cm petri dishes in DMEM complete medium for 3 days. Subsequently, cells were trypsinized, washed with PBS, and cell pellets fixed in ice-cold 70% ethanol for 2 hours at 4°C. The cells were then stained with propidium iodide (PI) solution (0.1% Triton X-100, 10 μg/ml PI, and 100 μg/ml DNase-free RNase A) for 30 minutes at room temperature and analyzed with the BD FACSAria II (BD Biosciences). Data were analyzed using FlowJo software (version 7.6.1). For intratumoral macrophage analysis, mice were left untreated or drug treated for 6 days starting on day 5 following B16-OVA tumor cell inoculation. Tumors were analyzed on day 12 or 13. For intratumoral analyses following adoptive OT-I T cell transfers, mice were drug treated from days 5–10 after tumor inoculation, followed by adoptive transfer on day 11 and FACS analysis on day 13 or 14. Tumors were harvested in FACS buffer (1% FBS in PBS) and digested in 2.5 ml/0.1 g tumor of 100 U/ml collagenase IV, 0.5 mg/ml DNase I (both Worthington Biochemical) in PBS. Erythrocytes were removed by 1-minute incubation in ACK lysis buffer (0.15 M NH_4_Cl, 10 mM KHCO_3_, 0.1 mM EDTA in PBS). Cell suspensions were stained with Zombie UV (BioLegend) for 15 minutes at room temperature for live cell detection, and 5 × 10^6^ cells were blocked with Fc-block (CD16/CD32, clone 2.4G2, Bio X Cell) for 15 minutes on ice and subsequently stained for 30 minutes on ice in FACS buffer with appropriate cell-surface antibodies (see Supplemental Table 2). The True-Nuclear Transcription Factor Buffer Set (BioLegend) was used for all intracellular staining. After 2 washes in permeabilization buffer, cells were analyzed using the BD FACSAria II (BD Biosciences) and DIVA software (version 6.1.3) (BD Biosciences). For all samples, 50,000–500,000 live singlets were analyzed. To FACS sort tumor-associated macrophages, tumor tissue was digested as described above. Erythrocytes were removed with ACK lysis buffer. Dead cells were excluded with Zombie UV stain (BioLegend). Cells were blocked with Fc block (Bio X Cell), followed by antibody staining. CD45^+^Gr1^–^CD11b^+^F4/80^+^ macrophages were sorted into ice-cold RPMI 1640 (Gibco, Thermo Fisher Scientific) containing 10% FBS. After sorting, macrophages were lysed with QIAzol (QIAGEN) and mRNA extracted for quantitative PCR (qPCR) analysis.

### Quantitative PCR analysis.

Tumors or cells were immediately snap-frozen in liquid nitrogen and RNA extracted using QIAzol (QIAGEN). cDNA was synthesized using VILO Superscript technology (Life Technologies). qPCR was performed using Rotor-Gene SYBR Green Master Mix (QIAGEN) and the Real Time PCR Detection System (QIAGEN). Each sample was run in duplicate qPCR reactions. Hypoxanthine-guanine phosphoribosyltransferase (*HPRT*) was used to calculate relative mRNA expression. The following primers were used: *HPRT*: forward primer (FP): ACACCTGCTAATTTTACTGGCAACA, reverse primer (RP): TGGAAAAGCCAAATACAAAGCCTA; *MMP9*: FP: GGACCCGAAGCGGACATTG, RP: GAAGGGATACCCGTCTCCGT; *RGS5*: FP: GCTTTGACTTGGCCCAGAAA, RP: CCTGACCAGATGACTACTTGATTAGCT; and *VEGFA*: FP: TGTACCTCCACCATGCCAAGT, RP: TGGAAGATGTCCACCAGGGT.

### Single-cell sequencing.

Tumors from 27-week-old RIP1-Tag5 mice were harvested and digested as previously described (24). Briefly, tumors were placed in FACS buffer (PBS, 1% FBS) on ice and minced using a scalpel. Tumors were digested under slow rotation in PBS containing 0.8 mg/ml dispase (Invitrogen), 0.2 mg/ml collagenase P (Roche), and 0.1 mg/ml DNAse I (Worthington Biochemical) at 37°C for 20 minutes. Digestion was stopped with FBS, and cells were resuspended in FACS buffer and viability assessed. Single-cell libraries were constructed from pooled tumors from 3 RIP1-Tag5 mice using 10x Chromium 3′, version 2, chemistry following the manufacturer’s protocol (10x Genomics). 10× Single-cell libraries were sequenced at the Australian Genome Research Facility on a S2 flow cell using a NovaSeq 6000 sequencer (Illumina). scRNA-Seq libraries were processed using Cell Ranger 2.1.1 with mm10-2.1.0 reference. Raw gene-barcode matrices from Cell Ranger output were used for downstream processing. Cells were distinguished from background noise using EmptyDrops (66). Outlier cells with a high ratio of number of detected unique molecular identifiers (UMIs) to genes (>3 median absolute deviations from median) were removed using Scater (67). Cells with fewer than 500 genes were excluded. Seurat, version 2, was used for sample integration and analysis (68).

### Organ slice culture.

Fresh meningioma specimens were collected and placed into ice-cold transfer medium (DMEM, Gibco, Thermo Fisher Scientific). Tumors were cut into cubes of approximately 1 × 1 × 1 cm^3^ dimensions. Tumor fragments were embedded in 4% low melting agarose in 1× PBS (NuSieve GTG, Lonza Bioscience) and placed on ice. Agarose-tumor cubes were submerged in ice-cold cutting media (140 mM NaCL, 5 mM KCL, 2 mM NaHCO_3_, 1 mM NaHPO_4_, 1.2 mM MgCL_2_, 1.5 mM CaCL_2_, 3 mM glucose, 10 mM HEPES pH 7.4) and cut into 300 μm thick slices using a Vibratome (Leica VT1200S, 2.8 mm amplitude, 0.6 mm/s speed). In a 24-well plate, each slice was placed centrally on top of a sponge (Ethicon, Spongostan, 1 cm^3^) presoaked in pericyte growth medium supplemented with 10% FCS and pericyte growth serum (all ScienCell Research Laboratories) with or without the addition of 50 mM trametinib. Sponge cultures were maintained in a 37°C incubator with 5% CO_2_ for 3–5 days. Medium was changed daily starting from day 2. Agarose was removed prior to embedding tumor slices into OCT for snap freezing. IHC was performed as described. For vessel quantification, 4 to 7 fields of ×20 images per tissue section were analyzed.

### Bioinformatic analyses.

Data were analyzed from a study investigating the clinical outcomes of anti-PD1 checkpoint inhibition in metastatic melanoma patients (43). Clinical data were downloaded from Supplemental Table 1 of this study (https://github.com/vanallenlab/schadendorf-pd1/blob/master/data/Supplemental_Table_1_wAge.tsv, commit ID 536010a). RNA-Seq data were located under https://github.com/vanallenlab/schadendorf-pd1/blob/master/data/addData.zip, commit ID d9df562. Whole-transcriptome sequencing data from formalin-fixed, paraffin-embedded tissue specimens were aligned using STAR and quantified using RNA-Seq by expectation-maximization (RSEM) to yield gene-level expected counts. Count data were normalized using edgeR’s Trimmed Mean of M-values (version 3.40.2) (69). Data from a total of 114 patients were analyzed (*n* = 54 alive, *n* = 60 dead) for metastatic disease progression. A gene signature for contractile markers was generated from vascular markers identified in this study and our previous publication (24). The contractile gene signature was composed of the following genes: *ACTG2*, *ACTA2*, *CNN1*, *CALD1*, *MYLK*, *MYH11*, *MYOCD*, and *CDH5*. Overall survival was used as the primary prognosis endpoint. For survival analysis, GSVA R package (version 1.46.0) was used to calculate an enrichment score for the contractile gene signature using a Gaussian kernel function (suitable for continuous expression data) (70) comparing top and bottom quartiles of the melanoma dataset. Survival outcomes of high- and low-expression signatures were compared by log-rank tests and plotted as Kaplan-Meier curves using the Survminer R package (version 0.4.9).

### Statistics.

GraphPad Prism software (version 10.1.2) was used for statistical analyses. Data are represented as mean ± SEM. Numbers (*n*) of mice or biological replica and *P* values are shown in figure legends. For comparison of groups, 1-way ANOVA with post hoc Tukey’s testing (unless otherwise indicated) or 2-tailed, unpaired Student’s *t* tests were used as indicated in figure legends. Tumor growth curves were analyzed using multiple unpaired *t* tests. Survival data were analyzed using log-rank (Mantel-Cox) tests. R, version 4.2.2, was used for bioinformatic analyses. *P* < 0.05 was considered significant.

### Study approval.

All animal studies were approved by the University of Western Australia (ET0000455, ET0000492) or the Harry Perkins Institute of Medical Research (AE077, AE261) animal ethics committees. All human studies were approved by the Sir Charles Gairdner Group and St. John of God Health Care Human Research ethics committees, Western Australia, Australia (RGS0000000919). Written, informed patient consent was received prior to sample collections.

### Data availability.

Mouse tumor scRNA-Seq data have been deposited in the NCBI’s Gene Expression Omnibus database (GEO GSE271508). Values for all data points in graphs are reported in the Supporting Data Values file. Other data generated in this study are available upon request.

## Author contributions

ZJL, BH, and AD are co–first authors and contributed equal numbers of figures/data. ZJL appears first because he developed cruical methodology. BH appears second because he contributed essential long-term study data. AD appears third because of a difference in overall time commitment. RG, ZJL, and BH conceived the project. ZJL, BH, AD, SS, RC, and JS developed methodology. DDL, JL, ZJL, BH, AD, RC, AAB, ED, KHW, AJP, ERB, and JS performed experiments. RG, ZJL, BH, and AD wrote the original draft of the manuscript. All authors contributed to reviewing and editing the manuscript. RG, GJH, BH, and JAN acquired funding. GYFL, GJH, LMN, and JAN provided resources. RG, JAN, GJH, and ARRF supervised the project.

## Supplementary Material

Supplemental data

Unedited blot and gel images

Supporting data values

## Version 1 09/17/2024

Electronic publication
