# Supplementary material for: Pericyte phenotype switching alleviates immunosuppression and sensitizes vascularized tumors to immunotherapy in preclinical models
Source: J Clin Invest. 2024 Sep 17;134(18):e179860. doi: 10.1172/JCI179860 (PMC11405053; doi:10.1172/JCI179860)
Supplement: Supplemental data [file jci-134-179860-s236.pdf]

# Supplemental Information

# Pericyte Phenotype Switching Alleviates Immunosuppression and Sensitizes Vascularized Tumors to Immunotherapy in Preclinical Models

**Li et al.**

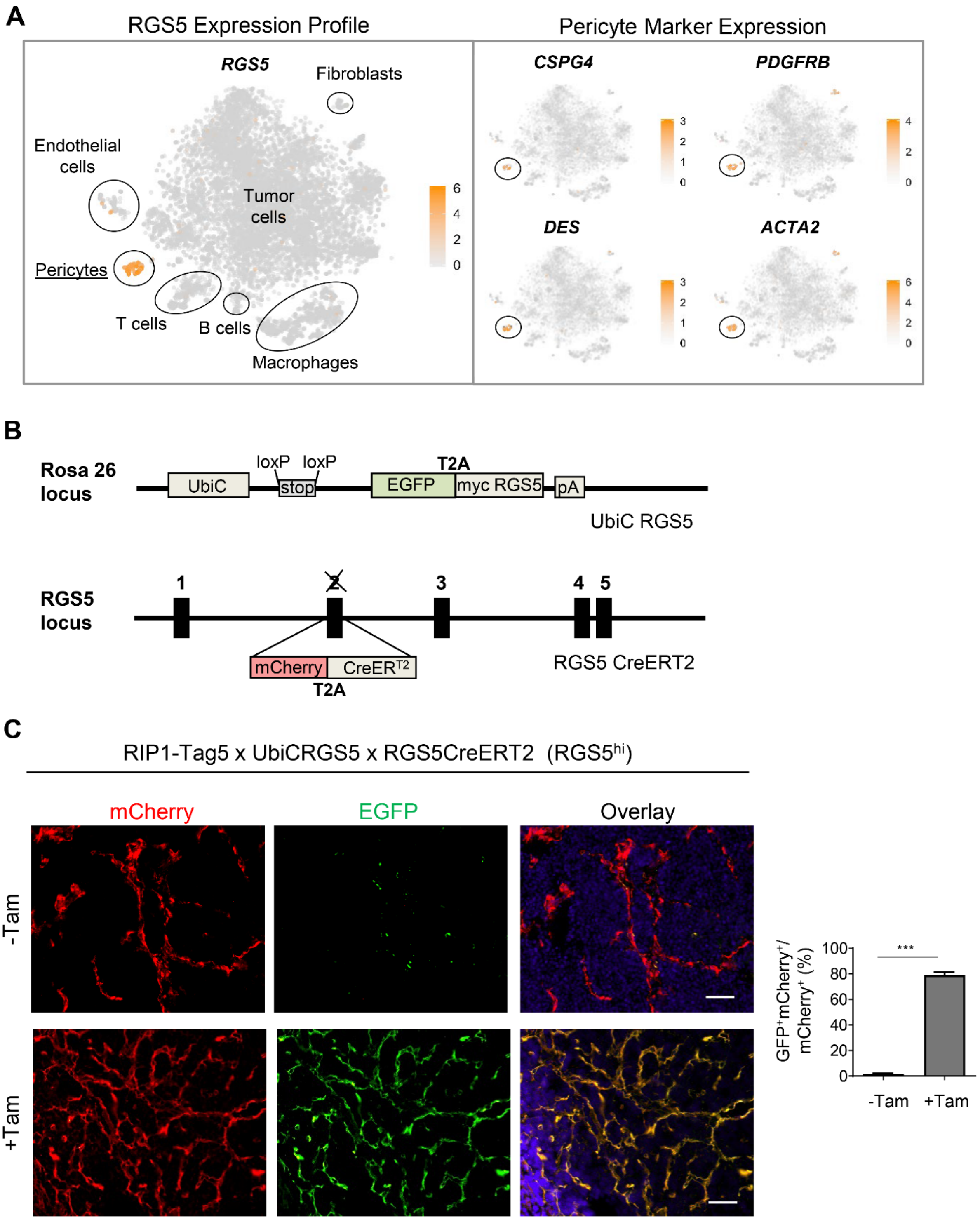
A
RGS5 Expression Profile
Pericyte Marker Expression
RGS5
Fibroblasts
Endothelial cells
Tumor cells
Pericytes
T cells
B cells
Macrophages
CSPG4
PDGFRB
DES
ACTA2
B
Rosa 26 locus
UbiC
loxP
stop
loxP
T2A
EGFP
myc RGS5
pA
UbiC RGS5
RGS5 locus
1
2
3
4
5
mCherry
CreER$^{T2}$
T2A
RGS5 CreERT2
C
RIP1-Tag5 x UbiCRGS5 x RGS5CreERT2 (RGS5$^{hi}$)
mCherry
EGFP
Overlay
-Tam
+Tam
GFP$^+$mCherry$^+$/ mCherry$^+$ (%)
***
-Tam
+Tam

**Supplemental Figure 1. RGS5 expression in PNET tumors is highly specific for pericytes and further upregulated in gain-of-function triple transgenic PNET.** (**A**) tSNE blots of single cell RNA sequencing data from RIP1-Tag5 wild type PNET tumors. Gene clustering locates *RGS5* mRNA expression to the pericyte cluster (orange, as defined by *CSPG4* (NG2), *PDGFRB*, desmin (*DES*) and αSMA (*ACTA2*) expression), but not tumor cells, endothelial cells, fibroblasts, immune cells or monocytes/macrophages, n=3 mice. (**B**) Schematic diagram of two knock-in mouse lines. Upper: insertion of the UbiC promoter, *EGFP* and myc-tagged *Rgs5* genes (separated by T2A peptide) into the Rosa26 mouse locus. Expression of transgenes is prevented by a loxP flanked stop cassette. Lower: *mCherry* and *CreERT*2 genes (separated by T2A) were inserted into exon 2 of the mouse *Rgs5* gene. *RGS5* overexpressing mice were generated by intercrossing the EGFP reporter line (UbiCRGS5) with the RGS5-Cre mCherry reporter mouse line (RGS5CreERT2). Further intercrossing of these 2 lines with RIP1-Tag5 (RIP1-Tag5 x UbiCRGS5 x RGS5CreERT2) generates triple transgenic, tumor-bearing mice which inherently express mCherry, and *EGFP/RGS5* driven by the endogenous *Rgs5* gene promoter upon tamoxifen induction ($Rgs5^{hi}$). (**C**) Representative histology of tumors in 27-week-old triple transgenic RIP1-Tag5 x UbiCRGS5 x RGS5CreERT2 mice before (-Tam, upper) and after tamoxifen (+ Tam, lower) injection at 24 weeks of age. Vascular expression of mCherry (red) and induction of vascular expression of EGFP (green) with tamoxifen are depicted. Quantification of $GFP^{+}$ $mCherry^{+}$ (yellow) vessels in relation to $mCherry^{+}$ vessels (red) approximates Cre recombination rate following tamoxifen induction, n=3 mice, mean ± SEM, ***P<0.0001. Student's t-test. Scale bars, 50 µm.

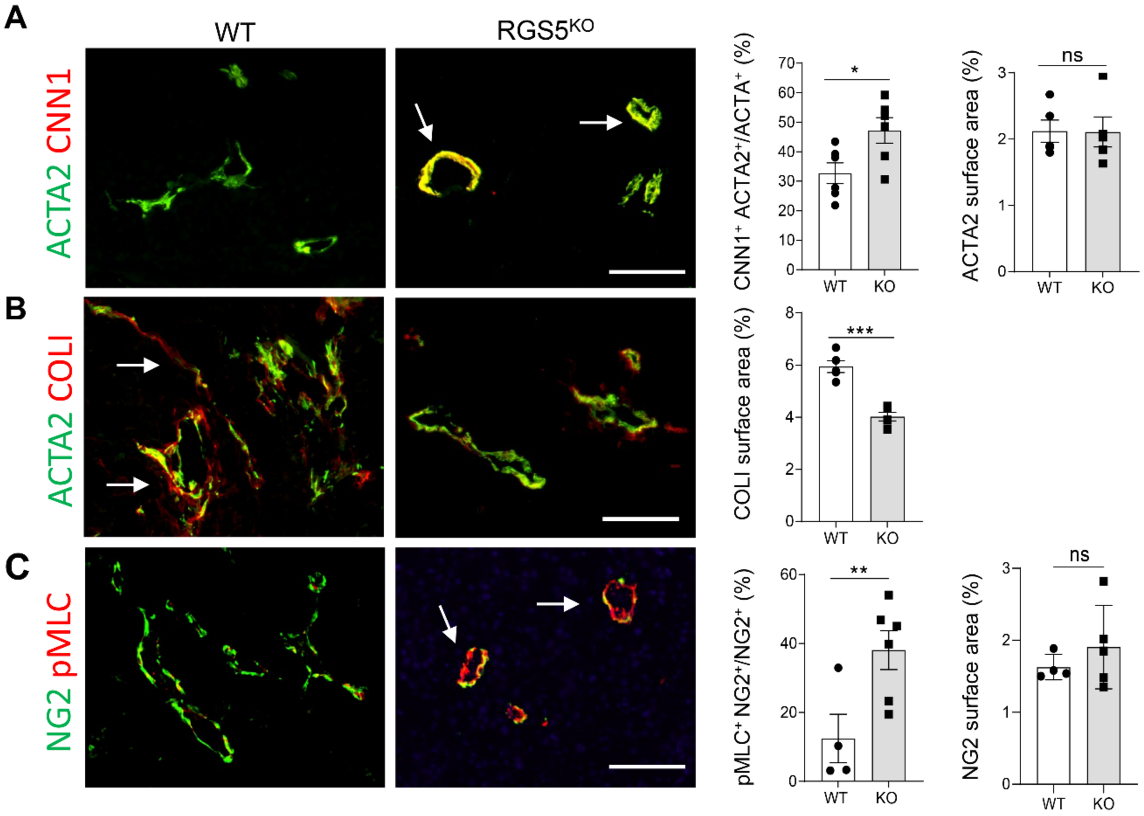


**Supplemental Figure 2. B16-OVA melanoma grown in *Rgs5*$^{KO}$ mice display an enhanced pericyte maturation status.** (**A**) B16-OVA tumors were grown in wild type (WT) or *Rgs5* knockout (*Rgs5*$^{KO}$ or KO) mice and CNN1 (red) coverage of ACTA2$^{+}$ pericytes (green) was quantified (overlay yellow), n=4-6 mice, *P=0.03, ns, not statistically significant, Students's *t*-test. Scale bar, 100 µm. (**B**) COLI (red) deposition around pericytes (ACTA2, green), n=4-6 mice, ***P=0.0001. Students's *t*-test. Scale bar, 100 µm. (**C**) pMLC (red) expression in pericytes (NG2, green), n=4-6 mice, **P=0.02, ns, not statistically significant, Students's *t*-test. Scale bar, 100 µm.

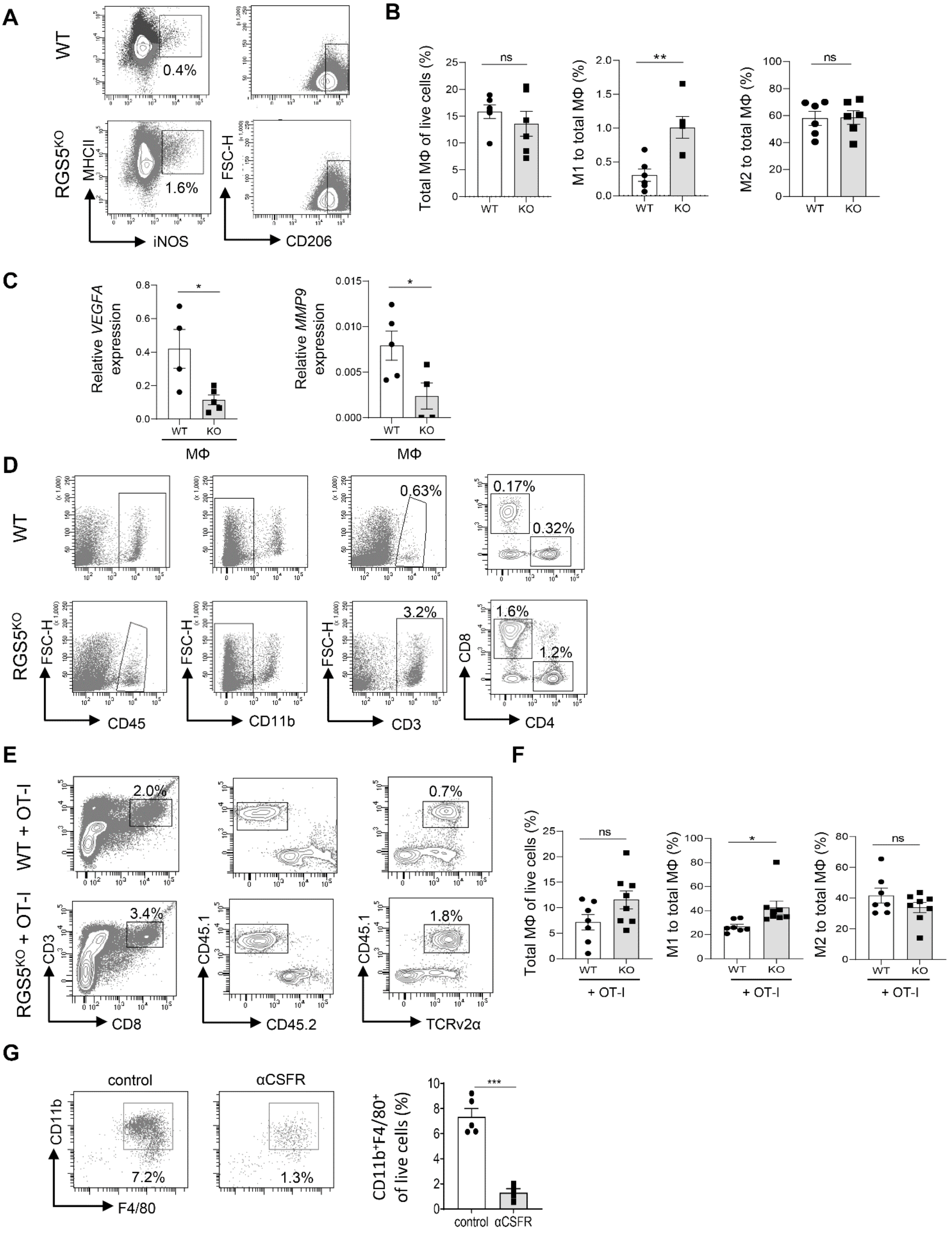

A
WT
RGS5KO
0.4%
1.6%
MHCII
iNOS
FSC-H
CD206
B
Total MΦ of live cells (%)
M1 to total MΦ (%)
M2 to total MΦ (%)
ns
**
ns
WT
KO
C
Relative VEGFA expression
Relative MMP9 expression
*
*
WT
KO
MΦ
D
0.63%
0.17%
0.32%
3.2%
1.6%
1.2%
FSC-H
CD45
CD11b
CD3
CD8
CD4
E
WT + OT-I
RGS5KO + OT-I
2.0%
3.4%
0.7%
1.8%
CD3
CD8
CD45.1
CD45.2
TCRv2α
F
Total MΦ of live cells (%)
M1 to total MΦ (%)
M2 to total MΦ (%)
ns
*
ns
+ OT-I
G
control
αCSFR
CD11b
F4/80
7.2%
1.3%
CD11b+F4/80+ of live cells (%)
***
control
αCSFR

**Supplemental Figure 3. Intratumoral immune cells in WT and *Rgs5*$^{KO}$ B16-OVA tumors change in numbers and characteristics.** (**A**) Representative FACS plots showing gating strategy for macrophage polarization in B16-OVA tumors from WT and *Rgs5*$^{KO}$ (KO) mice. Macrophages are characterized as CD45$^{+}$, Gr1$^{-}$, F4/80$^{+}$, CD11b$^{+}$, MHCII$^{high}$ (M1), iNOS$^{+}$ (M1) or CD206$^{high}$ (M2). (**B**) FACS quantification of total intratumoral macrophages (MΦ) gated on live cells, and percentage of M1 or M2 macrophages of total intratumoral macrophage populations in WT and *Rgs5*$^{KO}$ B16-OVA tumors, n=4-6 mice, mean ± SEM, **P=0.003, ns, not statistically significant, Student's *t*-test. (**C**) CD11b$^{+}$/F4/80$^{+}$ B16-OVA intratumoral macrophages were sorted by FACS and *VEGFA* and *MMP9* mRNA expression quantified by qPCR, n=4-5 mice, mean ± SEM, *P=0.04 (*VEGFA*), *P=0.03 (*MMP9*), Student's *t*-test. (**D**) Representative FACS plots showing gating strategy for intratumoral CD45$^{+}$, CD11b$^{+}$, CD3$^{+}$ CD4$^{+}$ or CD8$^{+}$ T cells in WT and *Rgs5*$^{KO}$ B16-OVA tumors. (**E**) Representative FACS plots showing gating strategy for congenic OT-I T cells, defined as CD3$^{+}$, CD8$^{+}$, CD45.2$^{-}$, CD45.1$^{+}$, TCRv2α$^{+}$ in WT and *Rgs5*$^{KO}$ B16-OVA tumors following OT-I T cell transfer. (**F**) FACS quantification of total intratumoral macrophages (MΦ) gated on live cells following adoptive OT-I transfer, and percentage of M1 or M2 macrophages of total macrophage populations following OT-I transfer, n=7-8 mice, mean ± SEM, *P=0.025, ns, not statistically significant, Student's *t*-test. (**G**) Representative FACS blots and quantification of CD11b$^{+}$, F4/80$^{+}$ intratumoral macrophages, gated on live cells, in untreated B16-OVA tumors (control), and tumors treated with macrophage-depleting αCSFR antibodies, n=4-5 mice, mean ± SEM, ***P=0.0001, Student's *t*-test.

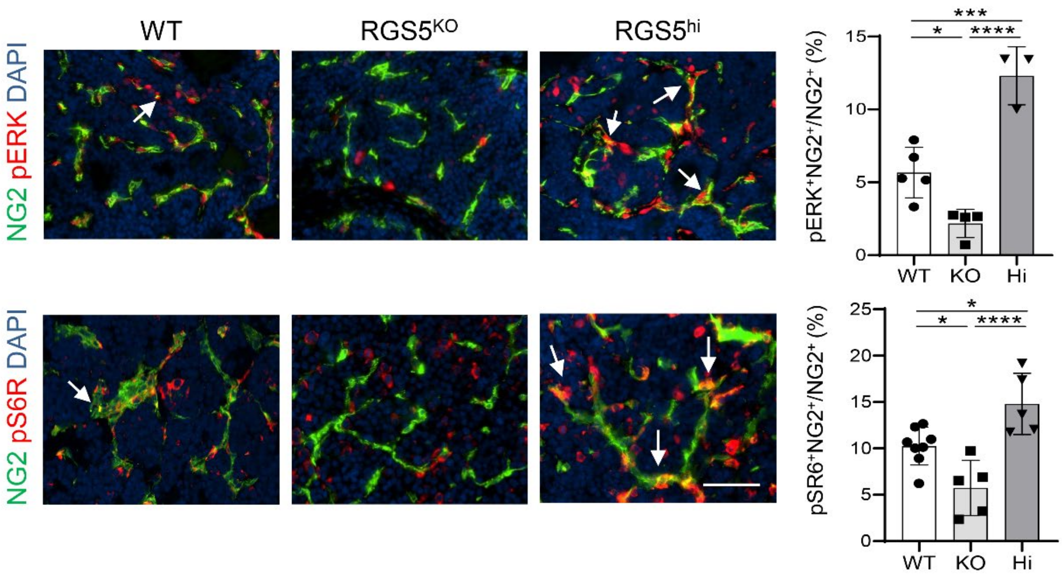


**Supplemental Figure 4. pERK and pS6R vascular expression correlates with RGS5 levels in PNET tumors.** Representative images from 27-week-old RIP1-Tag5 tumors grown in WT, *Rgs5*$^{KO}$ (KO), or *Rgs5*$^{hi}$ (Hi) mice depicting MEK/ERK signaling (pERK, red, upper) in NG2$^{+}$ pericytes (green), or AKT signaling (pS6R, red, lower) in NG2$^{+}$ pericytes (green), arrows indicate overlay (yellow). Quantification of vascular signaling: pERK, n=3-5 mice, mean ± SEM, *P=0.024, ***P=0.0008, ****P=<0.0001, one-way ANOVA. pS6R, n=5-8 mice, *P≤0.026, ****P=0.002, one-way ANOVA. Scale bar, 50 µm.

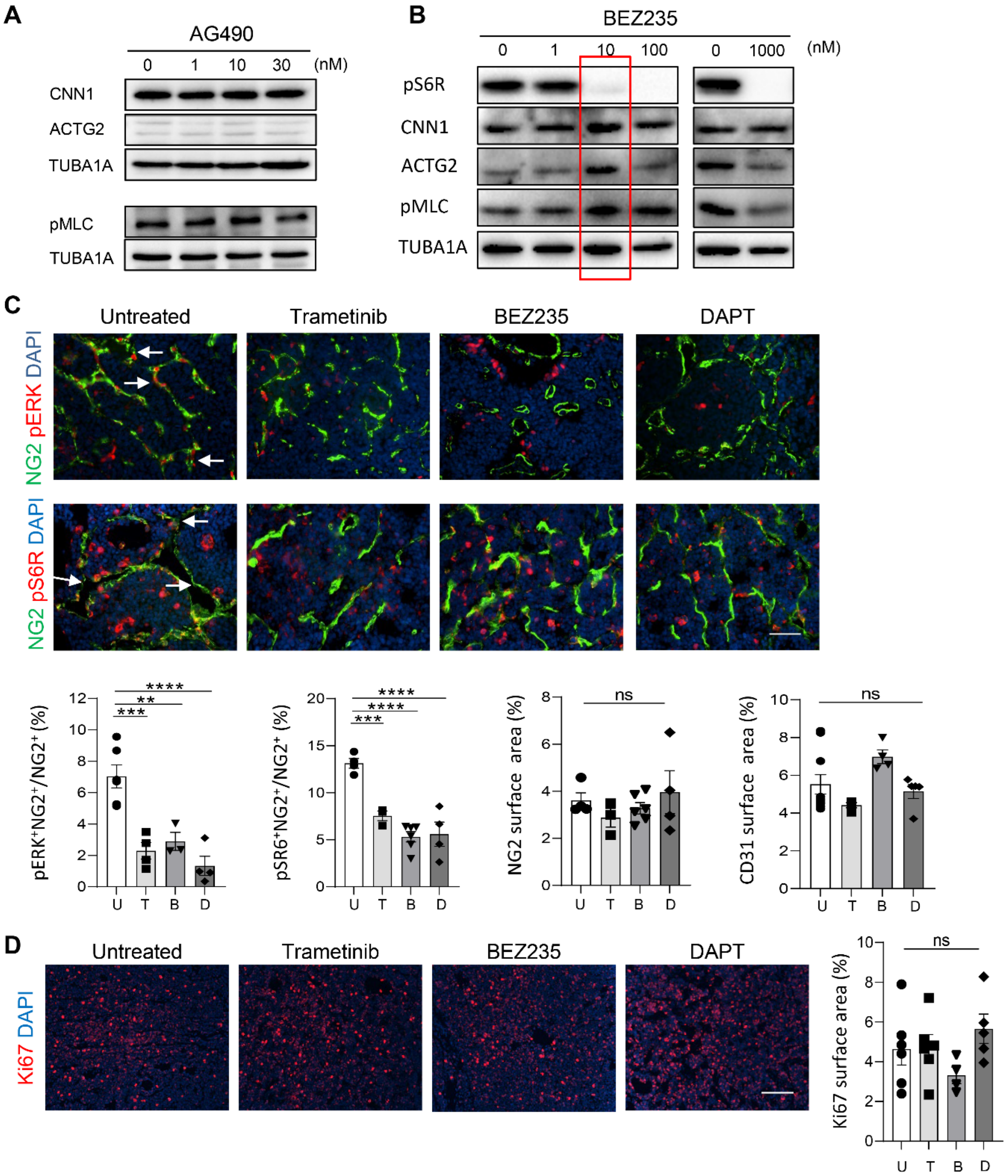

A
AG490
0 1 10 30 (nM)
CNN1
ACTG2
TUBA1A
pMLC
TUBA1A
B
BEZ235
0 1 10 100 0 1000 (nM)
pS6R
CNN1
ACTG2
pMLC
TUBA1A
C
Untreated
Trametinib
BEZ235
DAPT
NG2 pERK DAPI
NG2 pS6R DAPI
pERK+NG2+/NG2+ (%)
pSR6+NG2+/NG2+ (%)
NG2 surface area (%)
CD31 surface area (%)
****
**
***
****
****
***
ns
ns
U T B D
D
Untreated
Trametinib
BEZ235
DAPT
Ki67 DAPI
Ki67 surface area (%)
ns
U T B D

**Supplemental Figure 5. Contractile marker induction in vitro is dose dependent and pERK and pS6R vascular expression is reduced after low dose drug treatment.** (**A**) WB of contractile pericyte markers (CNN1, ACTG2) in correlation to Rho kinase activity (pMLC) in 10T1/2 RGS5myc cells following a 24 h incubation with increasing doses of AG490. The experiment was conducted twice. (**B**) Representative WB of AKT signaling (pS6R), contractile pericyte markers (CNN1, ACTG2) in correlation to Rho kinase activity (pMLC) in RGS5myc cells following a 24 h incubation with increasing doses of the PI3K inhibitor BEZ235. The experiment was conducted twice. (**C**) Representative images from untreated (U) RIP1-Tag5 mice, or following treatment from week 27 to 29 with trametinib (T, 0.02 mg/kg), BEZ235 (10 mg/kg), or DAPT (10 mg/kg) assessing vascular pERK (red, upper) or pS6R (red, lower) expression in pericytes (NG2, green). Arrows indicate overlay (yellow). Quantification of vascular signaling: pERK, **P=0.0053, ***P=0.0008, ****P=0.0001; pS6R: ***P=0.0015, ****P<0.0001; $NG2^{+}$ pericytes and $CD31^{+}$ blood vessels: ns, not statistically significant, n=3-6 mice, mean ± SEM, one-way ANOVA. Scale bar, 50 µm. (**D**) Tumor cell proliferation index following drug treatment in RIP1-Tag5 mice as assessed by Ki67 staining and quantification, n=4-6 mice, ns, not statistically significant, mean ± SEM, one-way ANOVA. Scale bar, 100 µm.

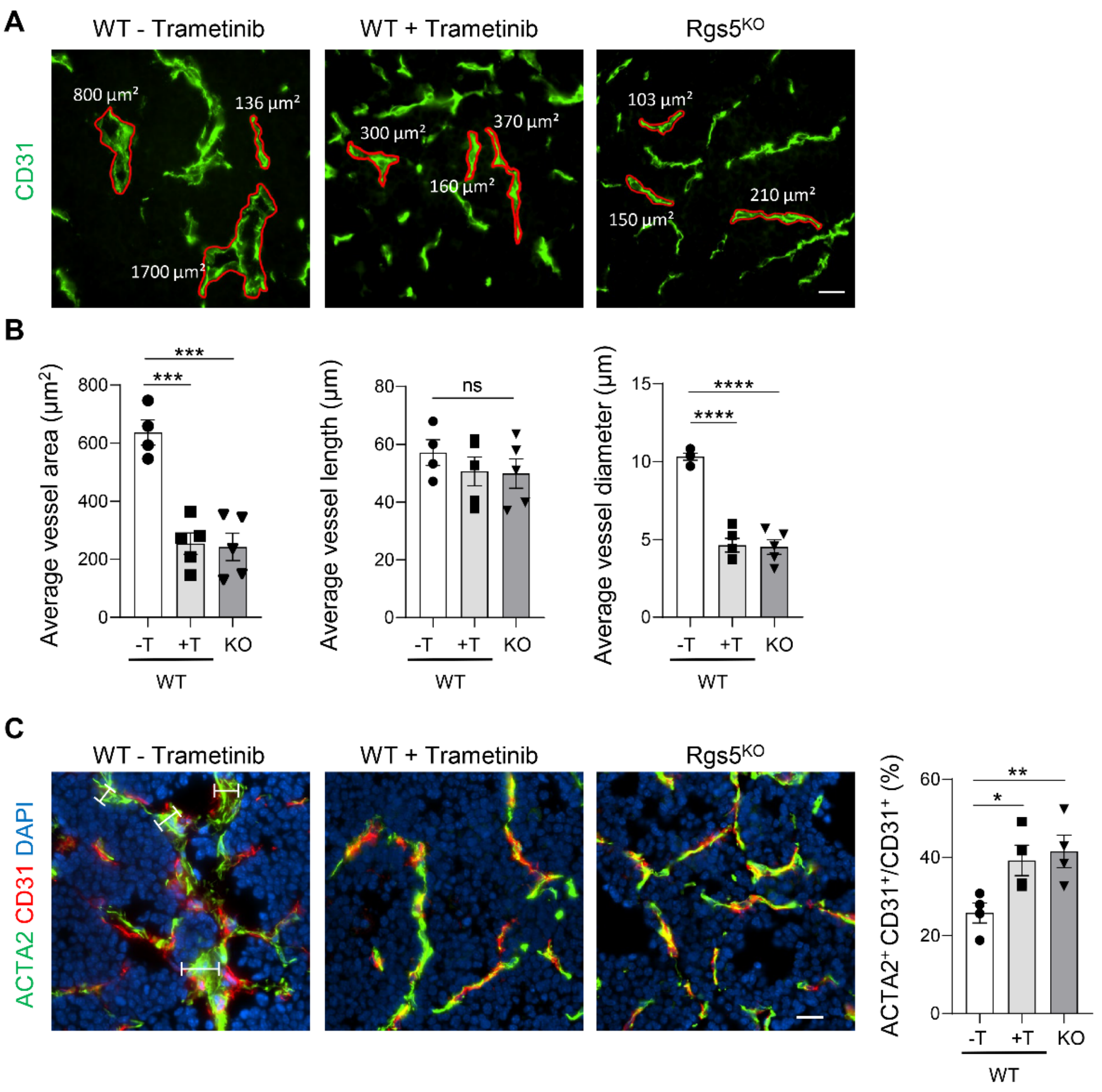


**Supplemental Figure 6. *Rgs5* gene knockout or trametinib treatment stabilize PNET tumor vessels.** (**A**) Untreated PNET-bearing RIP1-Tag5 mice (WT - T) or WT mice treated with trametinib for 2 weeks (WT + T), or $Rgs5^{KO}$ (KO) mice, were stained for $CD31^{+}$ tumor vessels (green) and average tumor vessel area demarcated in red. Scale bar, 20 µm. (**B**) Quantification of tumor vessel area (left), tumor vessel length (middle) and calculation of tumor diameters (right), n=4-5 mice, mean ± SEM, ***P=0.00001, ****P<0.00001, ns, not statistically significant, one-way ANOVA. (**C**) Same groups as in (**A**) were analzyed for $ACTA2^{+}$ (green) pericyte alignment with $CD31^{+}$ (red) endothelial cells and $ACTC2^{+}$ covered $CD31^{+}$ blood vessels quantified (yellow). Brackets indicate broad/fuzzy appearance of pericytes protrusions into parenchyma, n=4 mice, mean ± SEM, *P=0.0488, **P=0.0236, one-way ANOVA. Scale bar, 20 µm.

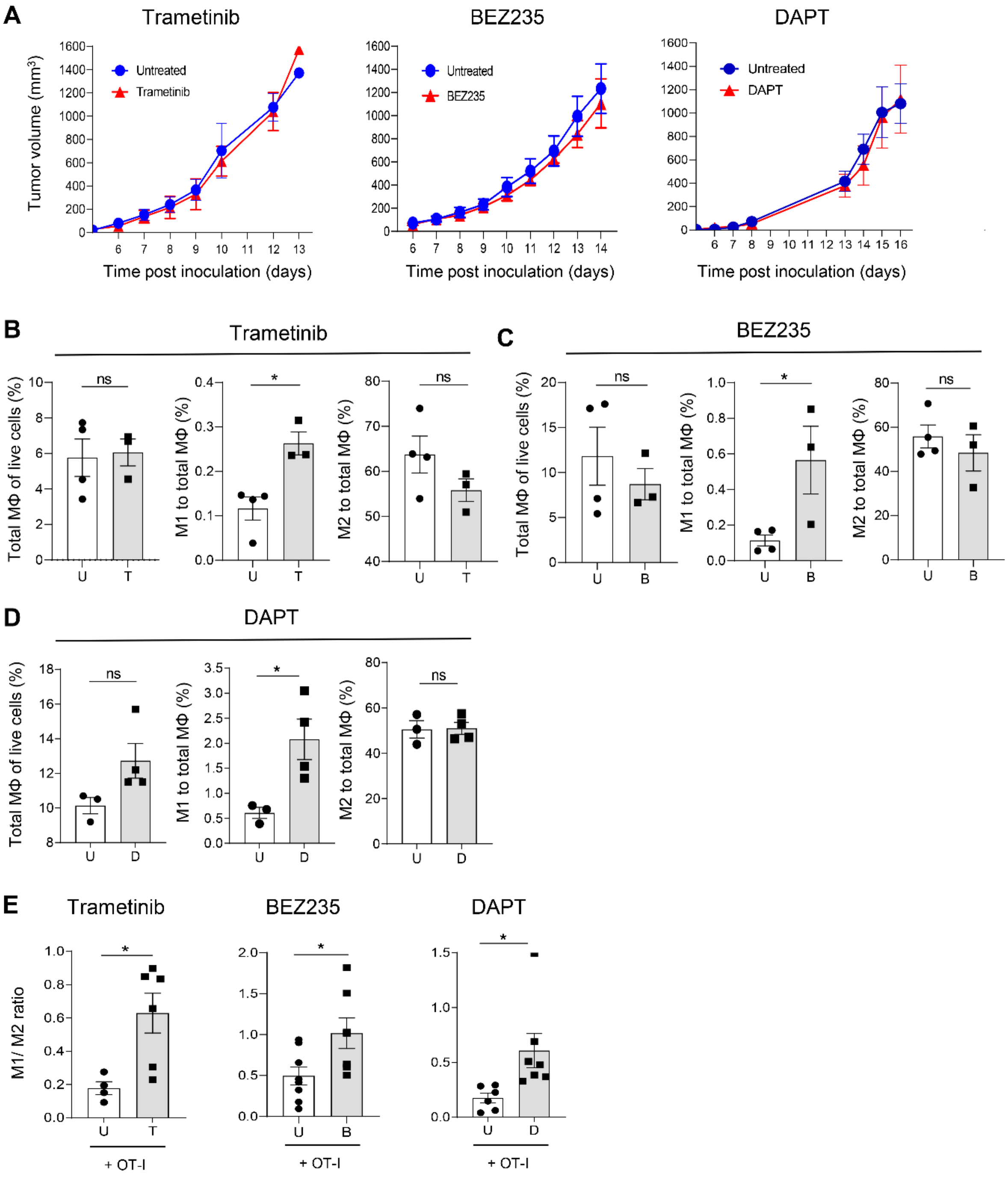
A
Trametinib
BEZ235
DAPT
Tumor volume (mm³)
Untreated
Trametinib
BEZ235
DAPT
Time post inoculation (days)
B
Trametinib
C
BEZ235
D
DAPT
Total MΦ of live cells (%)
M1 to total MΦ (%)
M2 to total MΦ (%)
ns
*
U
T
B
D
E
Trametinib
BEZ235
DAPT
M1/ M2 ratio
+ OT-I

**Supplemental Figure 7. Low dose drug treatment changes macrophage phenotypes but does not reduce tumor growth**. (**A**) Wild type mice bearing B16-OVA tumors were left untreated or treated from day 6 with 10 oral doses of trametinib (0.006 mg/kg, n=3-4 mice), BEZ235 (10 mg/kg, n=6-7 mice), or DAPT (10 mg/kg, n=6 mice), and tumor growth was monitored. (**B**) B16-OVA trametinib treatment: quantification of total intratumoral macrophages, gated on live cells, and percentage of M1 ($MHCII^{hi}$, $iNOS^{+}$) or M2 ($CD206^{high}$) macrophages in total intratumoral macrophage populations, n=3-4 mice, mean ± SEM, *P=0.0115, ns, statistically not significant, Student's *t*-test. (**C**) B16-OVA BEZ235 treatment: quantification of total intratumoral macrophages, gated on live cells, and percentage of M1 or M2 intratumoral macrophages in total macrophage populations, n=3-4 mice, mean ± SEM, *P=0.04, ns, statistically not significant, Student's *t*-test. (**D**) B16-OVA DAPT treatment: quantification of total intratumoral macrophages, gated on live cells, and percentage of M1 or M2 intratumoral macrophages in total macrophage populations, n=3-4 mice, mean ± SEM, *P=0.03, ns, statistically not significant, Student's *t*-test. **(E)** Intratumoral M1/M2 macrophage ratios in trametinib (left), BEZ235 (middle) or DAPT (right) treatment groups following one adaptive OT-I T cell transfer, n=3-5 mice, mean ± SEM, *P=0.02 (trametinib), *P=0.03 (BEZ235 and DAPT), Student's *t*-test.

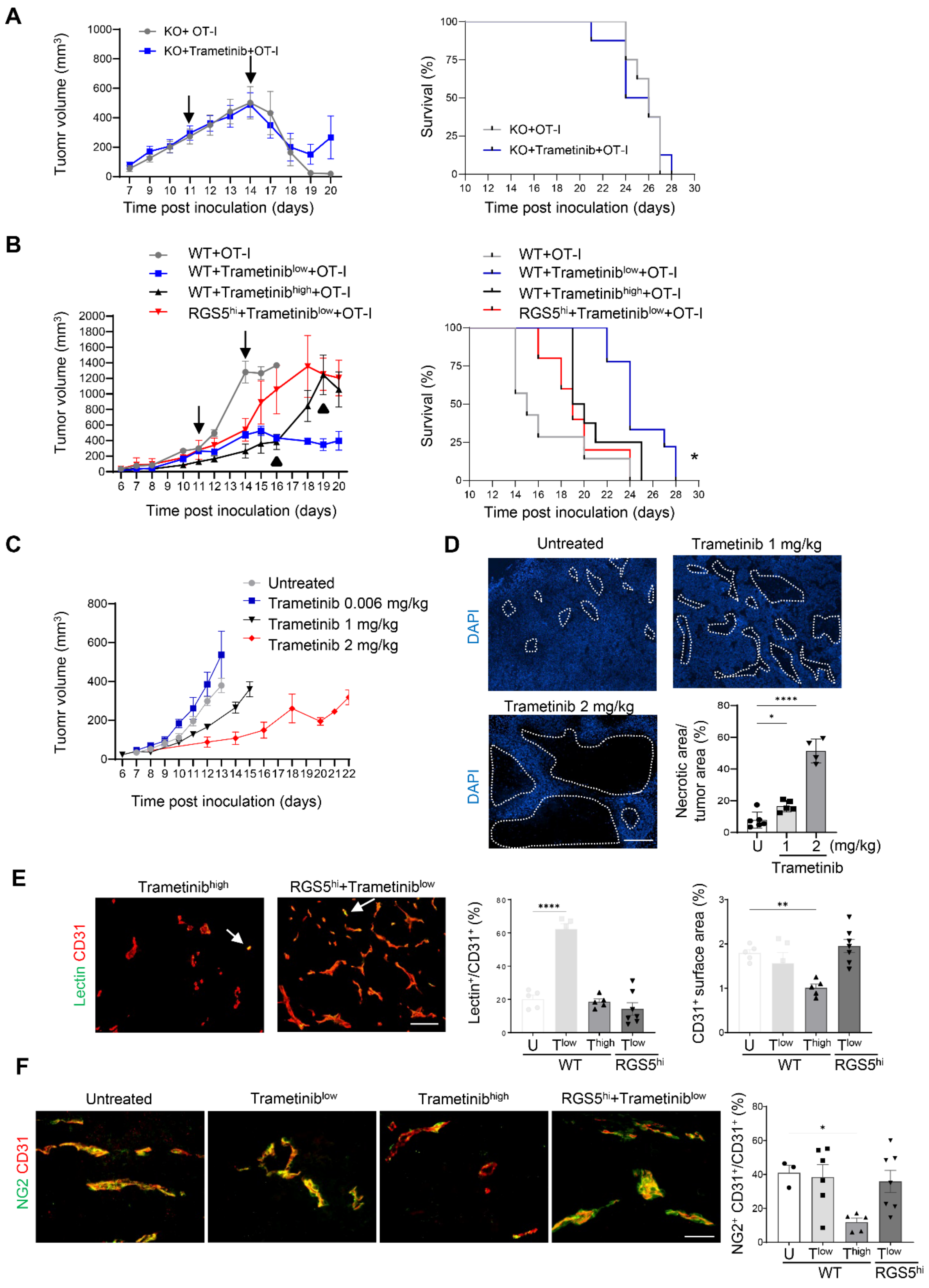
A
KO+ OT-I
KO+Trametinib+OT-I
Tuomr volume (mm³)
Time post inoculation (days)
Survival (%)
KO+OT-I
KO+Trametinib+OT-I
Time post inoculation (days)
B
WT+OT-I
WT+Trametinib^low+OT-I
WT+Trametinib^high+OT-I
RGS5^hi+Trametinib^low+OT-I
Tumor volume (mm³)
Time post inoculation (days)
Survival (%)
Time post inoculation (days)
C
Untreated
Trametinib 0.006 mg/kg
Trametinib 1 mg/kg
Trametinib 2 mg/kg
Tuomr volume (mm³)
Time post inoculation (days)
D
Untreated
Trametinib 1 mg/kg
Trametinib 2 mg/kg
DAPI
Necrotic area/ tumor area (%)
U 1 2 (mg/kg)
Trametinib
E
Trametinib^high
RGS5^hi+Trametinib^low
Lectin CD31
Lectin⁺/CD31⁺ (%)
CD31⁺ surface area (%)
U T^low T^high T^low
WT RGS5^hi
F
Untreated
Trametinib^low
Trametinib^high
RGS5^hi+Trametinib^low
NG2 CD31
NG2⁺ CD31⁺/CD31⁺ (%)
U T^low T^high T^low
WT RGS5^hi

**Supplemental Figure 8**. **Vessel functionality is critical for the success of low dose drug combination immunotherapy.** (**A**) *Rgs5*$^{KO}$ (KO) B16-OVA mice were treated with adoptive OT-I T cell transfers (arrows) with or without trametinib (0.006 mg/kg). Tumor growth and survival of n=8 mice, mean ± SEM. (**B**) WT or RGS5 overexpressing (*Rgs5*$^{hi}$) B16-OVA tumors received no drug or were treated with trametinib at different doses (Trametinib$^{low}$: 0.006 mg/kg; Trametinib$^{high}$: 1 mg/kg), followed by adoptive transfers of OT-I cells when tumor volume reached 300-400 mm$^3$ (indicated by arrows for all groups with the exception of WT/Trametinib$^{high}$ where arrow heads indicate delayed transfers). Tumor growth (n=5-10) and survival of n=5-9 mice, mean ± SEM. Survival was terminated on day 30, *P=0.001, WT Trametinib$^{low}$ versus WT no Trametinib; *P=0.02, WT Trametinib$^{low}$ versus WT Trametinib$^{high}$; *P=0.005, WT Trametinib$^{low}$ versus *Rgs5*$^{hi}$ Trametinib$^{low}$, log rank (Mantel-Cox) test. (**C**) Assessment of tumor growth kinetics in untreated compared to WT B16-OVA mice treated with increasing doses of trametinib, n=3-10 mice, mean ± SEM. (**D**) DAPI nuclear stain and quantification of B16-OVA tumor necrosis defined as absence of nuclear stain (dotted lines) following trametinib treatment, n=4-6, mean ± SEM, *P=0.035, ****P<0.0001, one-way ANOVA. Scale bar, 500 µm. (**E**) Assessment of tumor perfusion following trametinib treatments. CD31 (red) overlay with infused FITC-lectin (yellow) is highlighted by arrows. Perfusion and vessel numbers were quantified in WT B16-OVA mice treated with 1 mg/kg trametinib (T$^{high}$), or *Rgs5*$^{hi}$ B16-OVA mice treated with low dose trametinib (RGS5$^{hi}$ + T$^{low}$) in comparison to untreated and 0.006 mg/kg (T$^{low}$) treatment groups (data from Figure 5A, shadowed), n=5-7 mice, mean ± SEM, **P=0.0083, ****P<0.0001, one-way ANOVA. Scale bar, 100 µm. (**F**) Pericyte (NG2$^+$, green) vessel (CD31$^+$, red) coverage in B16-OVA treatment groups, n=3-7, *P=0.034, one-way ANOVA. Scale bar, 50 µm.

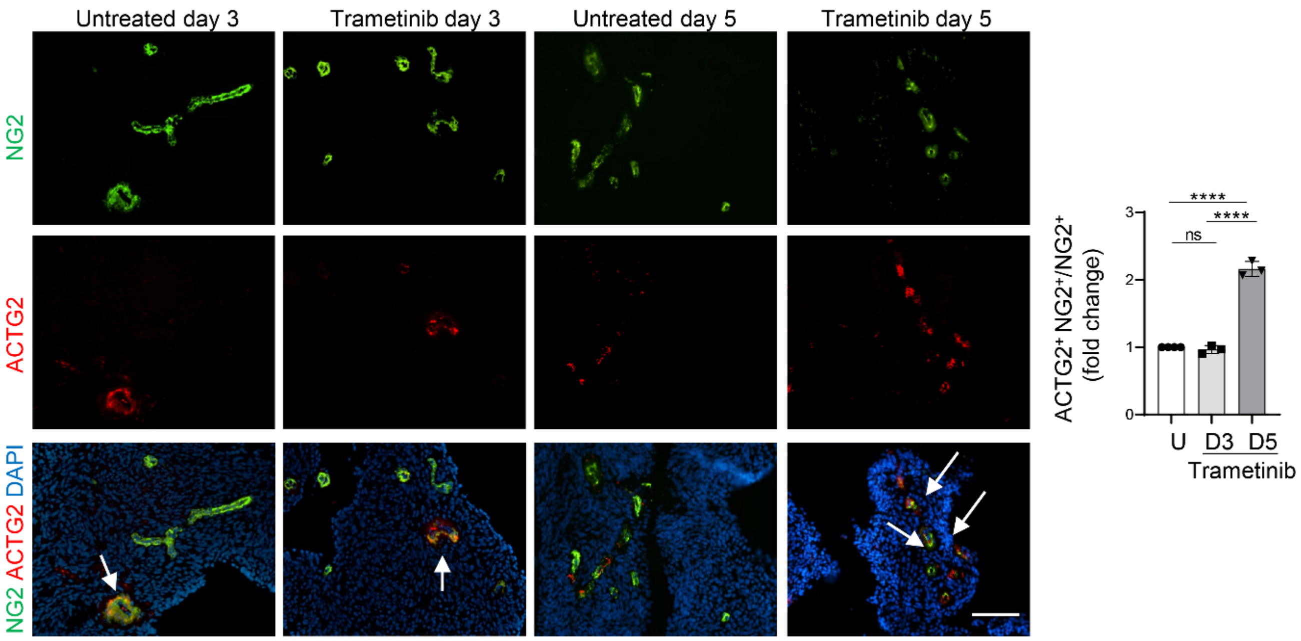


**Supplemental Figure 9**. **Trametinib induces the contractile marker ACTG2 in brain cancer pericytes**. Microscopic images of meningioma tumor slices cultured ex vivo for 3 or 5 days with or without trametinib. ACTG2 staining (red) depicts mature ACTG2$^+$ covered (yellow, arrows) NG2$^+$ (green) pericytes. Quantification of ACTG2 covered NG2$^+$ pericytes in untreated meningioma slices (U, day 3, day 5), and slices incubated with 50 mM trametinib for 3 and 5 days (D3, D5), n=3 patients, mean ± SEM, ****P<0.0001, ns, not statistically significant, one-way ANOVA. Scale bar, 100 µm.

**Table 1. Antibodies for Western Blot and Immunohistochemistry**

| Name | Host | Clone | Color | Catalogue # | Source | Antibody ID |
|---|---|---|---|---|---|---|
| | | | **Western blot** | | | |
| **ACTG2** | rabbit | polyclonal | - | NB100-91649 | Novus | AB_1216156 |
| **AKT (pan)** | rabbit | monoclonal | - | 4691 | Cell Signaling | AB_915783 |
| **pAKT (Ser 473)** | rabbit | D93 | - | 4060 | Cell Signaling | AB_2315049 |
| **CNN1** | rabbit | EP798Y | - | 46794 | Abcam | AB_2291941 |
| **CNX43** | rabbit | polyclonal | - | C6290 | Sigma | AB_476857 |
| **ERK1/2 (pan)** | rabbit | 137F5 | - | 4695 | Cell Signaling | AB_390779 |
| **pERK (Thr202/Tyr204)** | rabbit | D13.14.4E | - | 4376 | Cell Signaling | AB_2315112 |
| **Foxo3a (pan)** | rabbit | 75D8 | - | 2497 | Cell Signaling | AB_836876 |
| **pFoxo3a (Thr32/Thr24)** | rabbit | polyclonal | - | 9464S | Cell Signaling | AB_329842 |
| **KLF4 (H-180)** | rabbit | polyclonal | - | sc20691 | Santa Cruz | AB_669567 |
| **pMLC (Ser20)** | rabbit | polyclonal | - | ab2480 | Abcam | AB_303094 |
| **P27KIP1 (pan)** | rabbit | polyclonal | - | sc3674 | Santa Cruz | AB_632129 |
| **pP27KIP1 (Thr187)** | rabbit | polyclonal | - | sc16324-R | Santa Cruz | AB_670358 |
| **ROCK 1** | rabbit | polyclonal | - | 4035s | Cell Signaling | AB_2238679 |
| **ROCK2** | rabbit | polyclonal | - | 8236s | Cell Signaling | AB_10829468 |
| **ACTA2** | mouse | 14A | FITC | F3777 | Sigma | AB_476977 |
| **TUBA1A** | mouse | B-5-1-2 | - | T6074 | Sigma | AB_477582 |
| | | | **Immunohistochemistry** | | | |
| **ACTG2** | rabbit | polyclonal | - | AP06002PU | OriGene | AB_1610944 |
| **CD3e** | rabbit | polyclonal | - | 52959 | Abcam | AB_868901 |
| **CD31** | rat | MEC13.3 | - | 550274 | BD Biosciences | AB_393571 |
| **CD31** | rat | SZ31 | - | DIA-310 | Biozol | AB_2631039 |
| **CD31- biotin** | rat | MEC13.3 | - | 553371 | BD Biosciences | AB_394817 |
| **CDH5 (CD144)** | rat | 11D4.1 | - | 555289 | BD Biosciences | AB_395707 |
| **CNN1** | rabbit | EP7998Y | - | ab46794 | Abcam | AB_2291941 |
| **CALD1** | rabbit | E89 | - | ab32330 | Abcam | AB_725810 |
| **CNX43** | rabbit | polyclonal | - | C6290 | Sigma | AB_476857 |
| **COLI** | rabbit | polyclonal | - | PAB13488 | Abnova | AB_10556808 |
| **EGFP** | rabbit | polyclonal | - | GTX113617 | GeneTex | AB_1950371 |
| **pERK** | rabbit | D13.14.4E | - | 4370S | Cell Signaling | AB_2315112 |
| **Ki67** | rat | SolA15 | - | 14-5698-82 | Thermo Fisher | AB_10854564 |
| **mCherry** | mouse | 1C51 | - | ab125096 | Abcam | AB_11133266 |
| **pMLC (Ser20)** | rabbit | polyclonal | - | ab2480 | Abcam | AB_303094 |
| **NG2** | rabbit | polyclonal | - | AB5320 | Millipore | AB_91789 |
| **NG2** | rat | 1E6.4 | - | 130-097-455 | Miltenyi Biotec | AB_2651235 |
| **SMA, alpha** | mouse | 1A4 | FITC | 3777 | Sigma | AB_476977 |
| **pS6Rp** | rabbit | D57.2.2E | - | 4858S | Cell Signaling | AB_916156 |
| **ICAM** | hamster | 3E2B | - | MA5405 | Invitrogen | AB_223595 |

| Table 2. Antibodies for FACS and Secondary Antibodies | | | | | | |
|---|---|---|---|---|---|---|
| Name | Host | Clone | Color | Catalogue # | Source | Antibody ID |
| | | | **FACS** | | | |
| **CD3e** | **hamster** | eBio500A2 | PerCp-eFlour 710 | 46-0033-82 | Thermo Fisher | AB_10597122 |
| **CD3e** | **hamster** | 145-2C11 | FITC | 553062 | BD Biosciences | AB_394595 |
| **CD4** | **rat** | Gk1.5 | BUV737-APC | 100412 | BioLegend | AB_312697 |
| **CD8** | **rat** | 53-6-7 | PE | 553033 | BD Biosciences | AB_394571 |
| **CD11b** | **rat** | M1/70 | APC-Cy7 | 101226 | BioLegend | AB_830642 |
| **CD45** | **rat** | 30F-11 | PE-CF594 | 562420 | BD Biosciences | AB_11154401 |
| **CD45.1** | **mouse** | A20 | BUV737 | 564574 | BD Biosciences | AB_2738850 |
| **CD45.2** | **mouse** | 104 | PE-Cy7 | 560696 | BD Biosciences | AB_1727494 |
| **CD206** | **rat** | C068C2 | PE-Cy7 | 141720 | BioLegend | AB_2562248 |
| **F4/80** | **rat** | BM8 | APC | 123116 | BioLegend | AB_893481 |
| **Gr-1** | **rat** | RB6-8C5 | PE | 108408 | BioLegend | AB_313373 |
| **iNOS** | **rat** | CXNFT | AF-488 | 53-5920-82 | Thermo Fisher | AB_2574423 |
| **MHCclass II (I-A/I-E)** | **rat** | M5/114.15.2 | PerCP-Cy5.5 | 107626 | BioLegend | AB_2191071 |
| **TCRv2α** | **rat** | B20.1 | APC | 127810 | BioLegend | AB_1089250 |
| | | | **Secondary Antibodies** | | | |
| **anti-FITC** | **goat** | polyclonal | biotin | ab6655 | Abcam | AB_305628 |
| **anti-goat IgG** | **donkey** | polyclonal | AF488 | ab150129 | Abcam | AB_2687506 |
| **anti-hamster IgG** | **goat** | polyclonal | AF488 | A21110 | Thermo Fisher | AB_2535759 |
| **anti-hamster IgG** | **rabbit** | polyclonal | Cy3 | 307165003 | Jackson | AB_2339586 |
| **anti-mouse IgG** | **goat** | polyclonal | Dylight 405 | 115475003 | Jackson | AB_2338786 |
| **anti-mouse IgG** | **horse** | polyclonal | HRP | PI-2000 | Vector | AB_2336177 |
| **anti-rabbit IgG** | **donkey** | polyclonal | AF488 | A21206 | Thermo Fisher | AB_243579 |
| **anti-rabbit IgG** | **donkey** | polyclonal | AF594 | A21207 | Thermo Fisher | AB_141637 |
| **anti-rabbit IgG** | **goat** | polyclonal | HRP | PI-1000 | Vector | AB_1000 |
| **anti-rat IgG AF488** | **donkey** | polyclonal | AF488 | A21208 | Thermo Fisher | AB_2535794 |
| **anti-rat IgG** | **donkey** | polyclonal | AF459 | A21209 | Thermo Fisher | AB_2435795 |
| **Anti-rat IgG** | **donkey** | polyclonal | Dylight 405 | 712-475-153 | Jackson | AB_2340681 |
