# Supplementary material for: Pericyte phenotype switching alleviates immunosuppression and sensitizes vascularized tumors to immunotherapy in preclinical models
Source: J Clin Invest. 2024 Sep 17;134(18):e179860. doi: 10.1172/JCI179860 (PMC11405053; doi:10.1172/JCI179860)

Figure 1

E Experiment 1

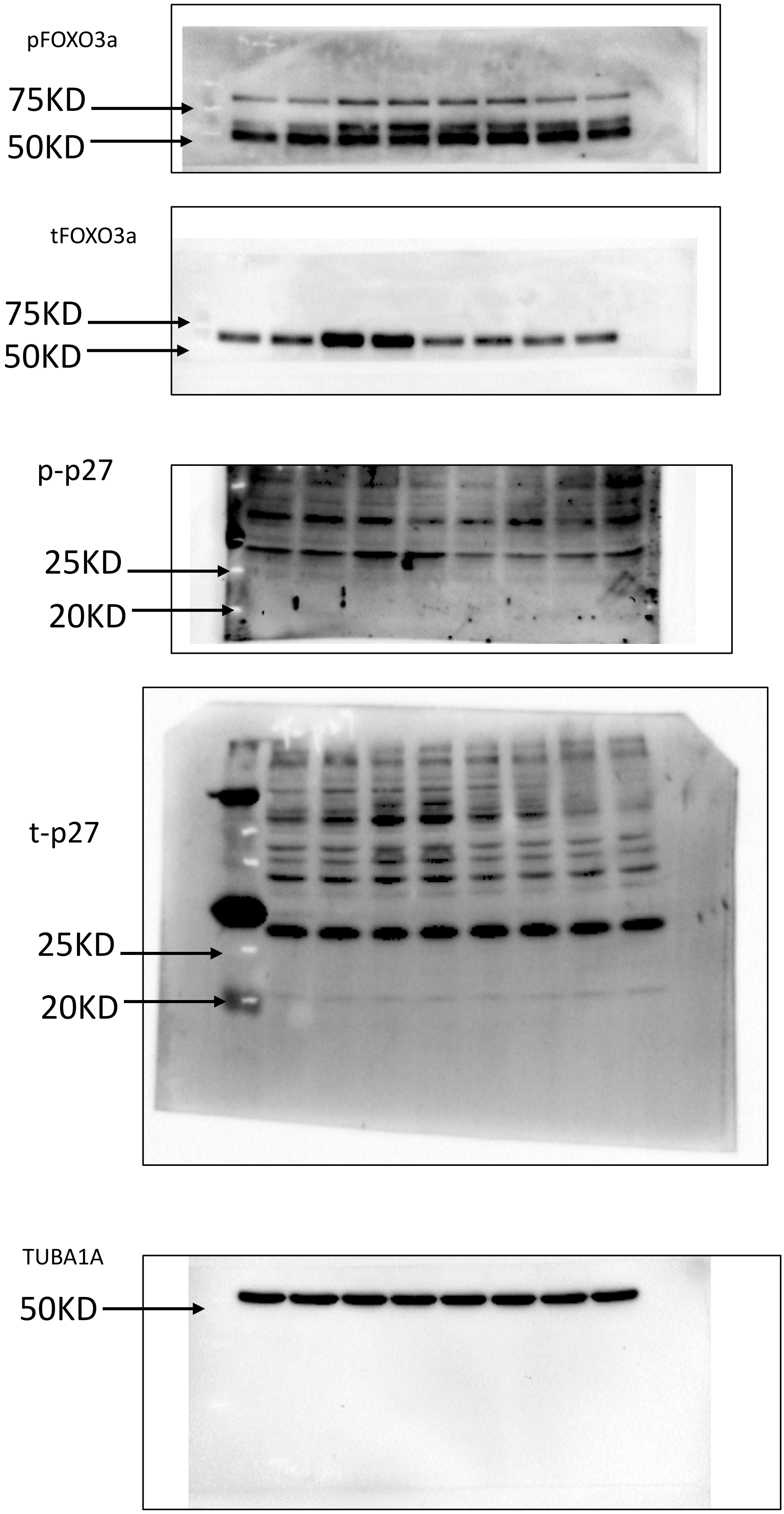

Experiment 2

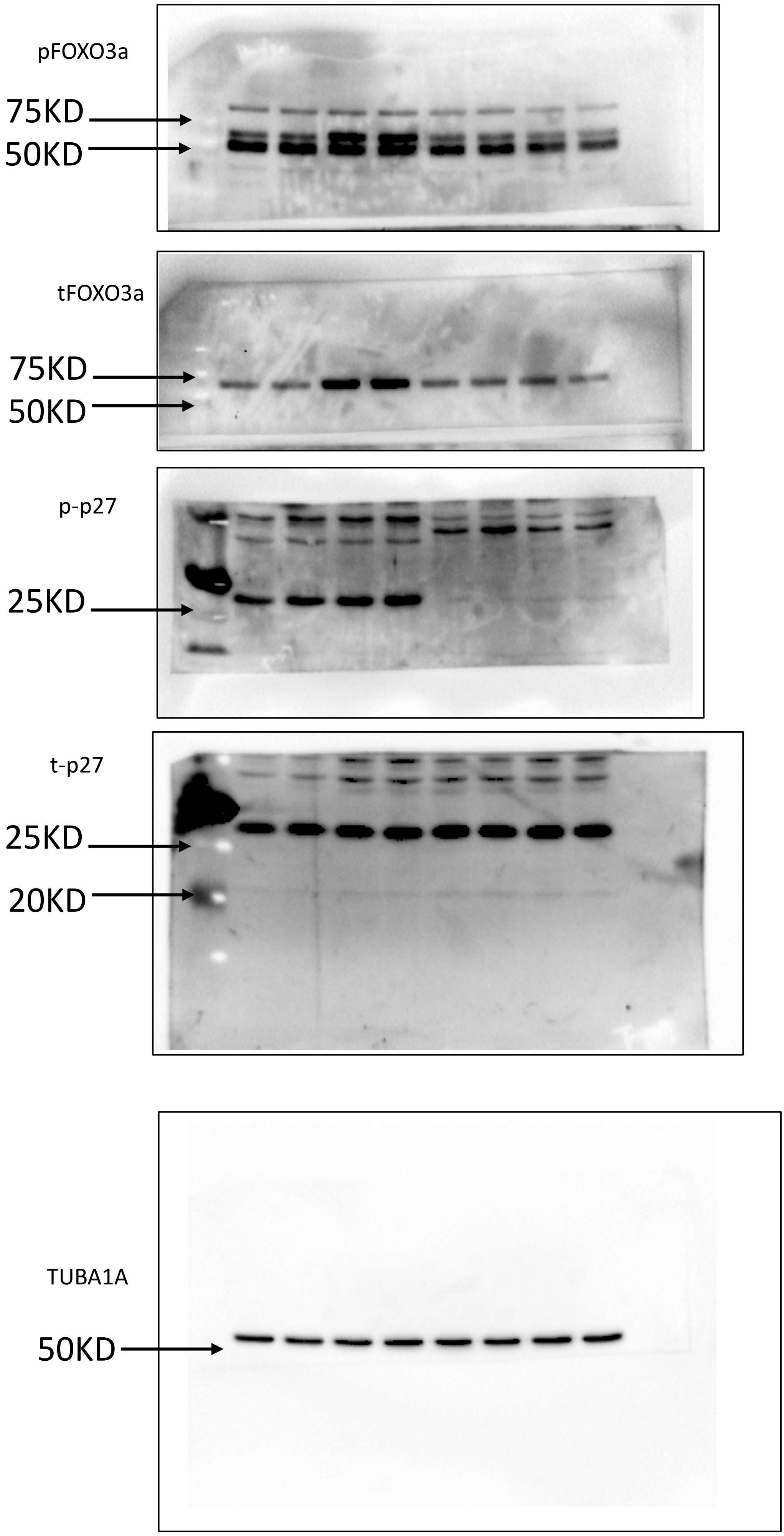

Experiment 3

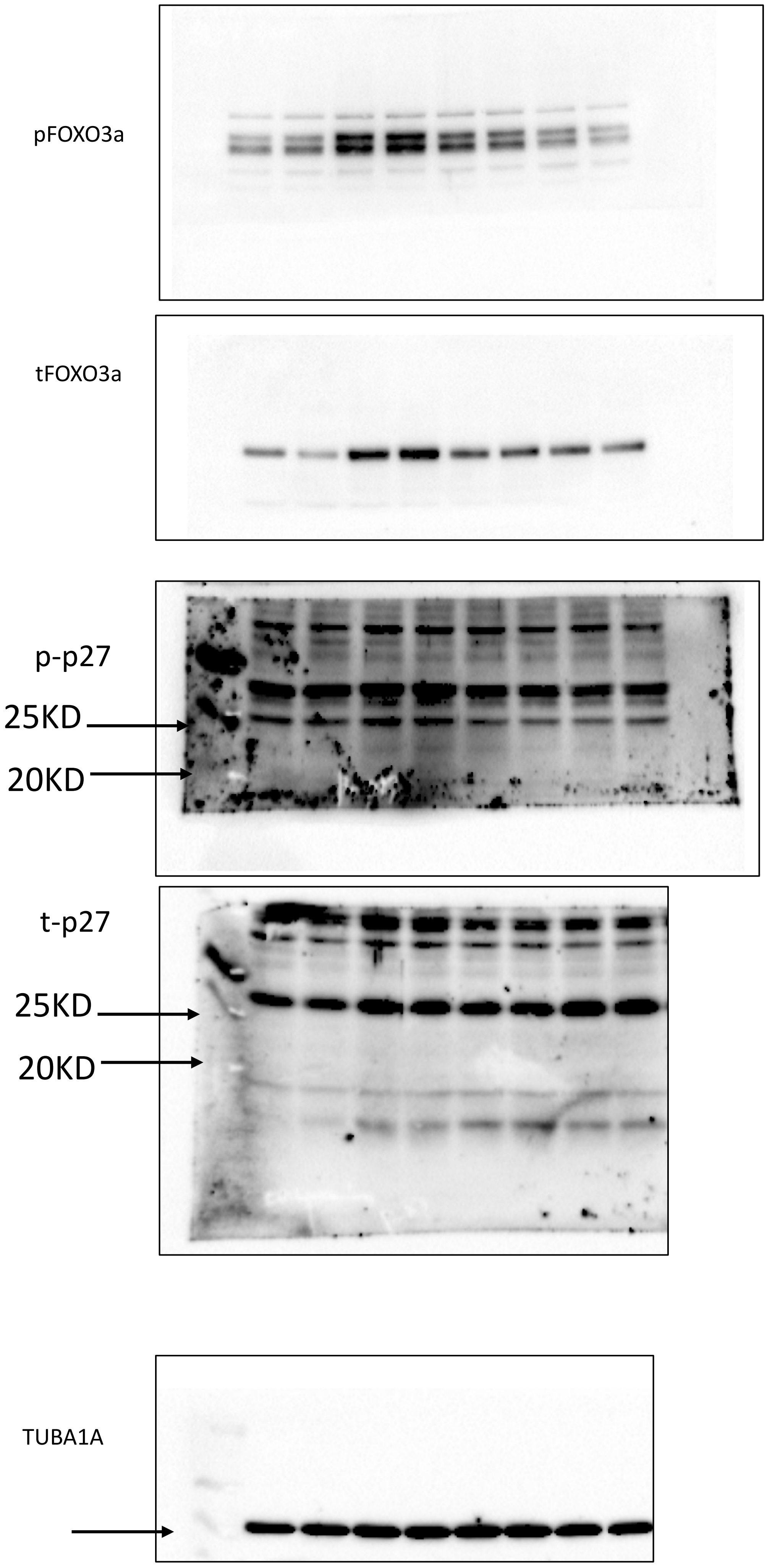

F

Experiment 1

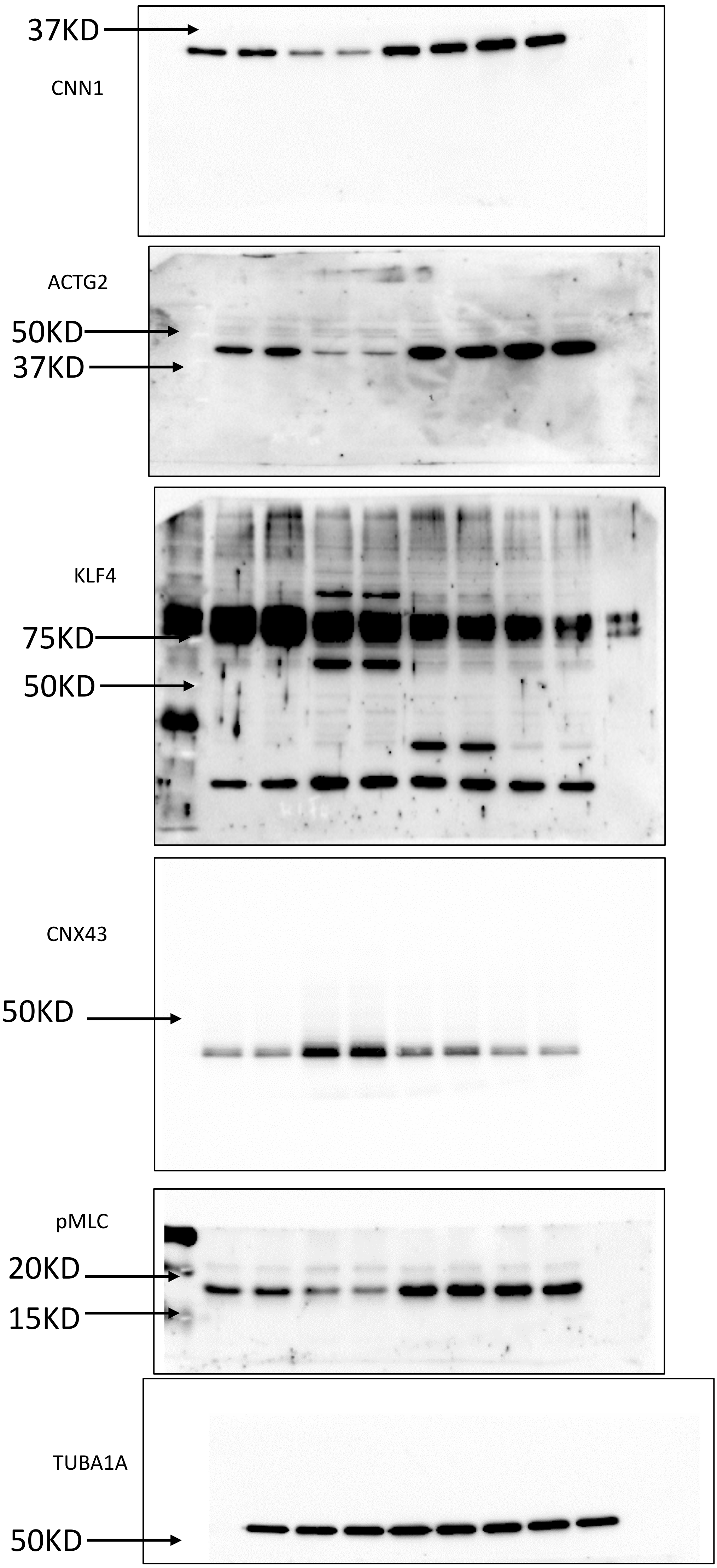

Experiment 2

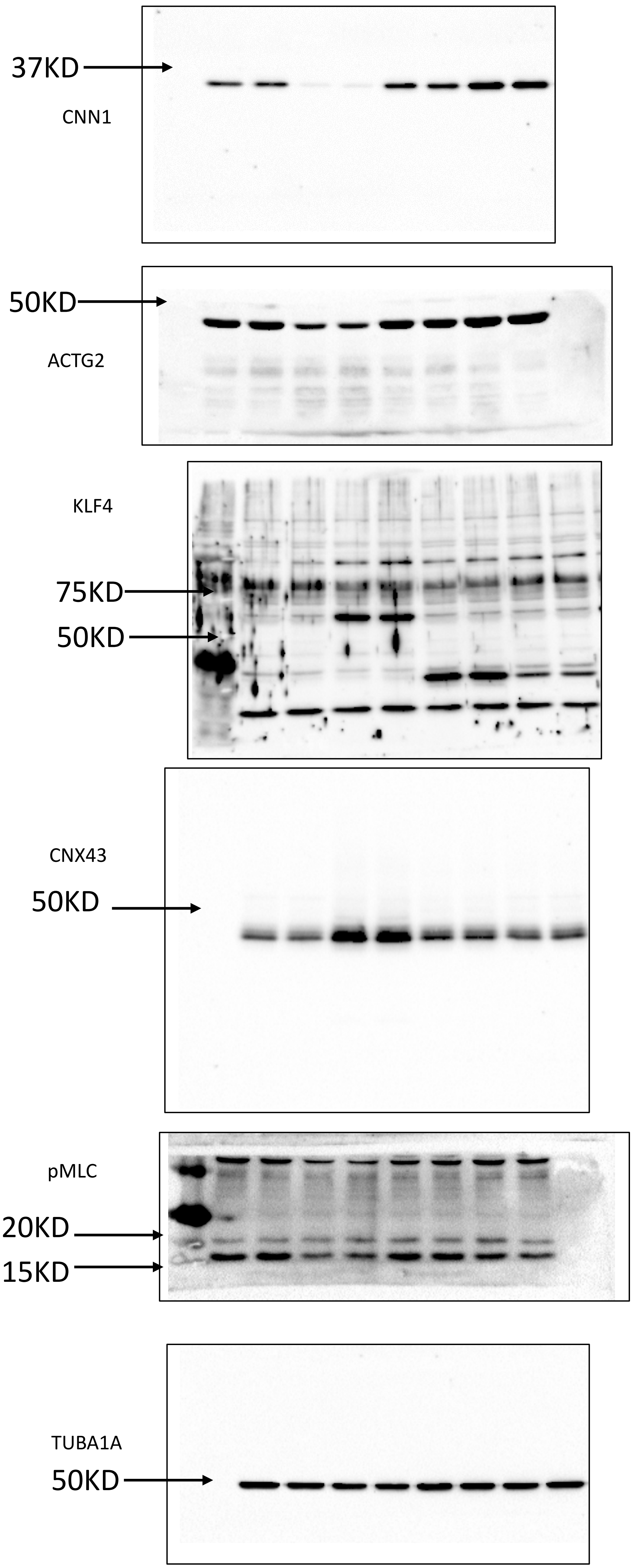

Experiment 3

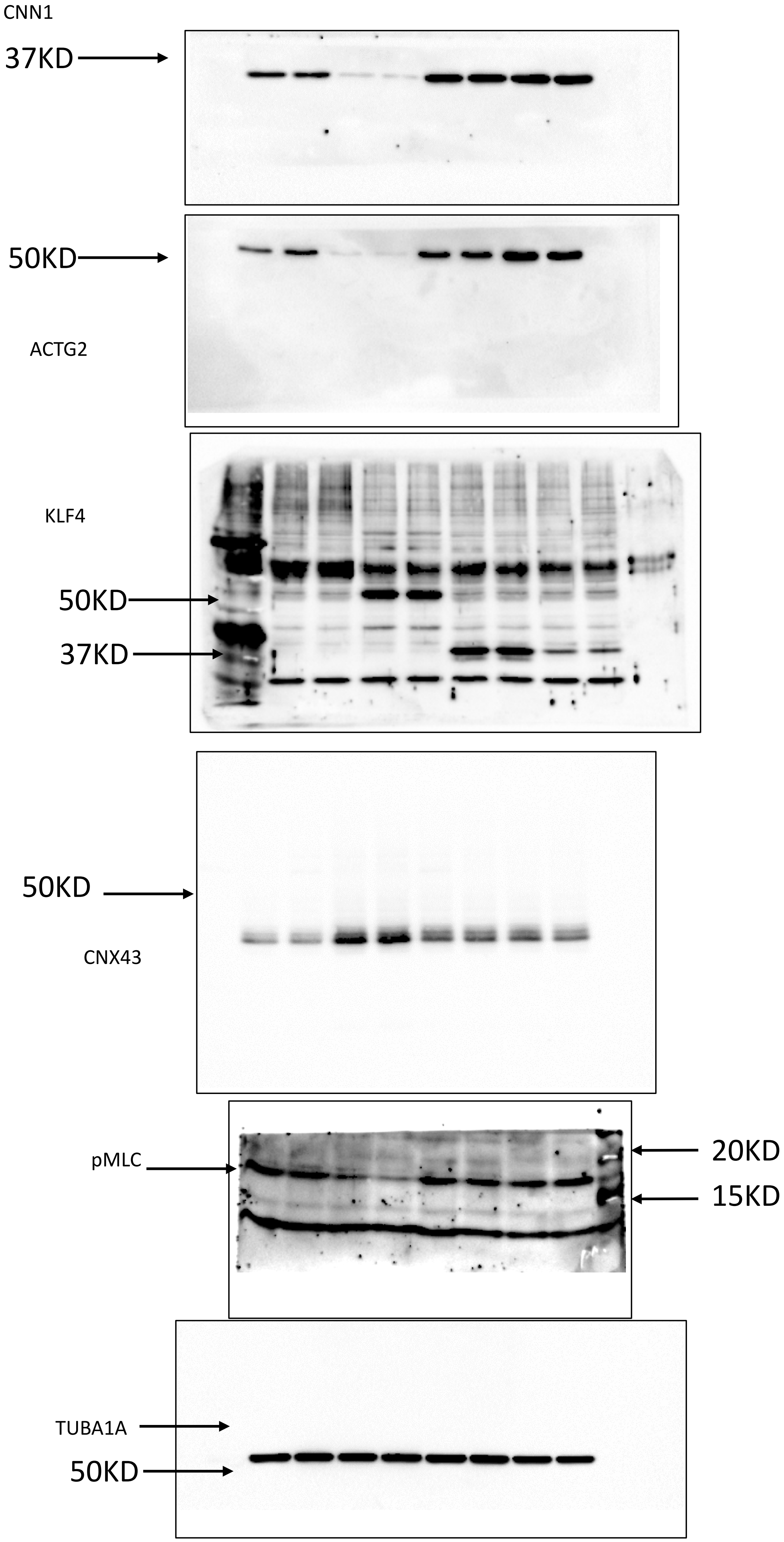

G

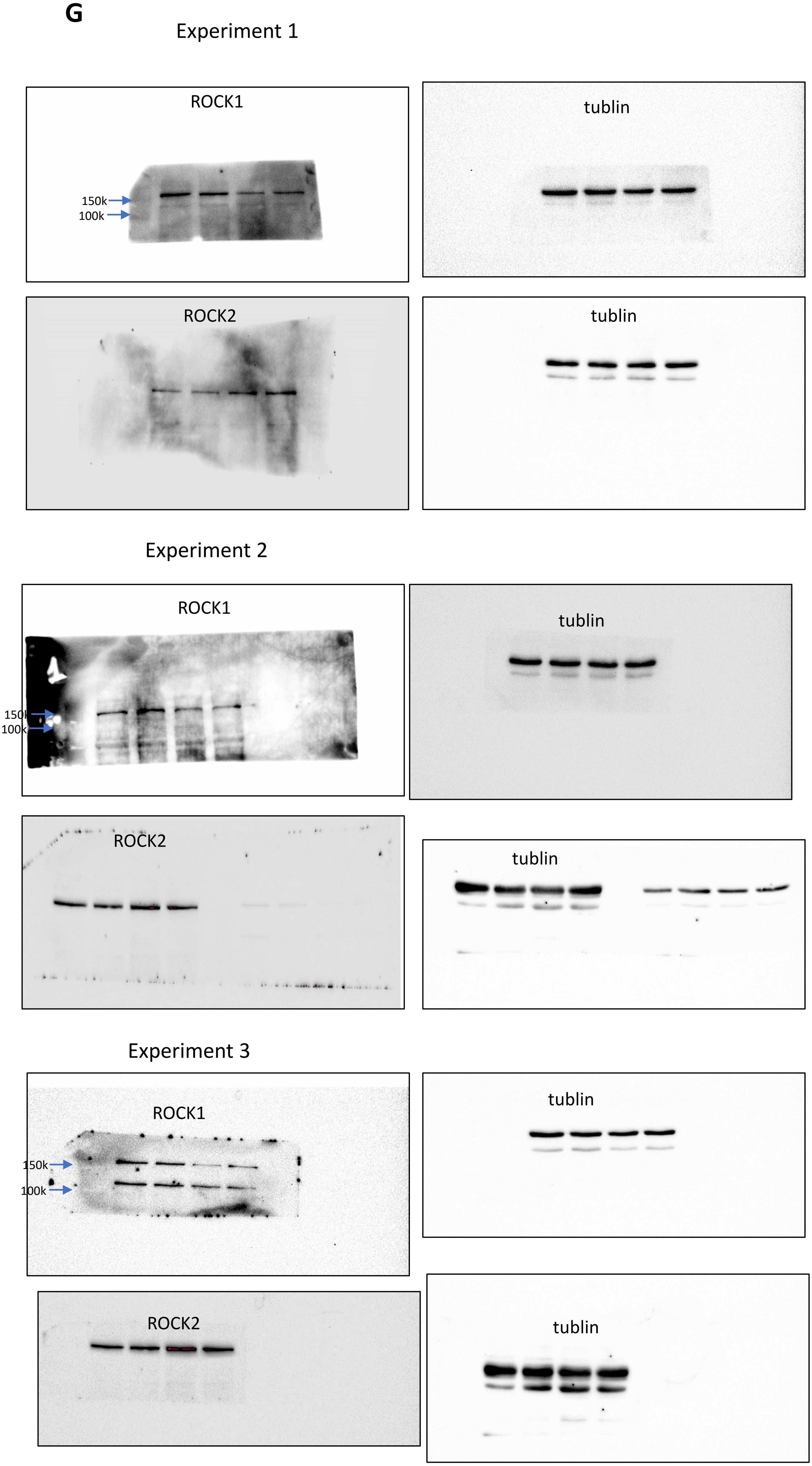
Experiment 1
ROCK1
150k
100k
tublin
ROCK2
tublin
Experiment 2
ROCK1
150k
100k
tublin
ROCK2
tublin
Experiment 3
ROCK1
150k
100k
tublin
ROCK2
tublin

Figure 4

D (Trametinib)

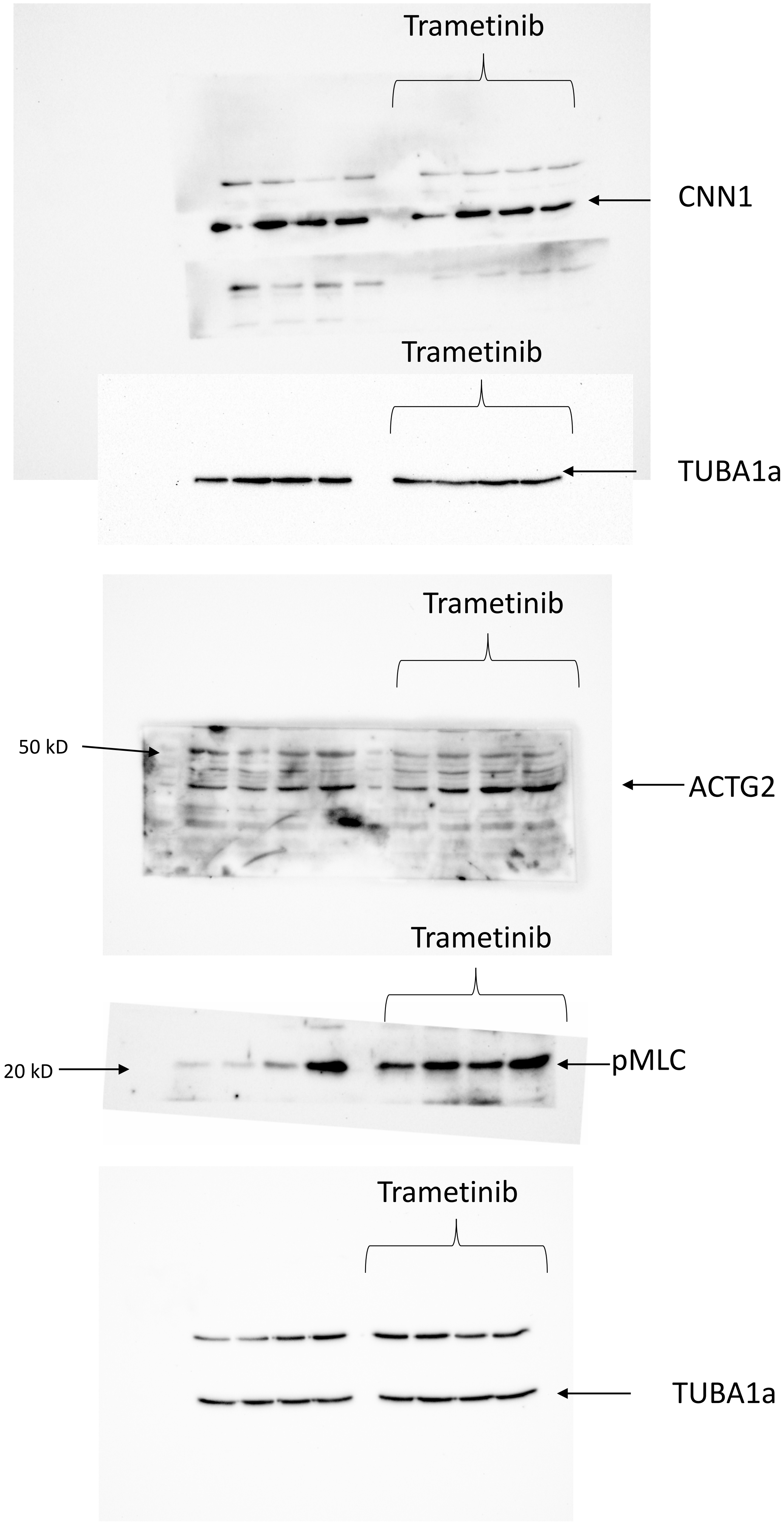

E (BEZ235)

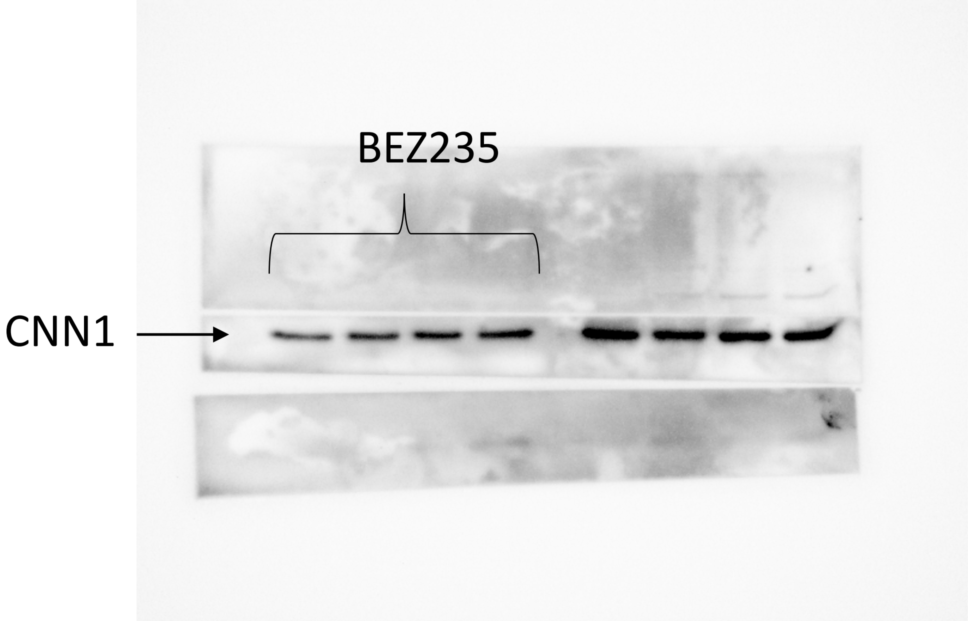

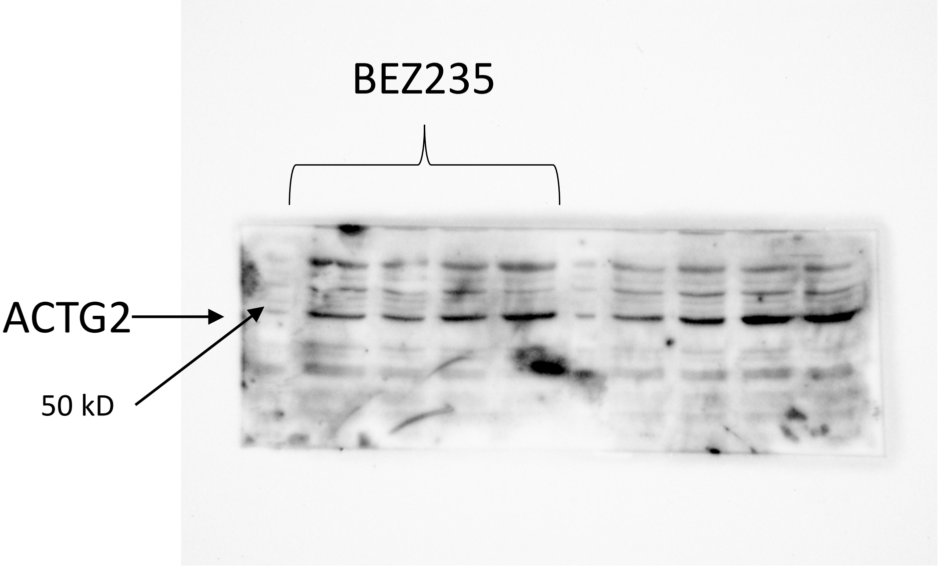

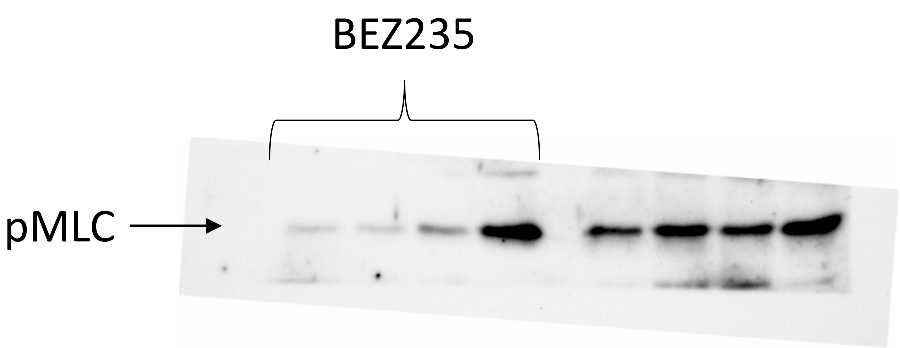

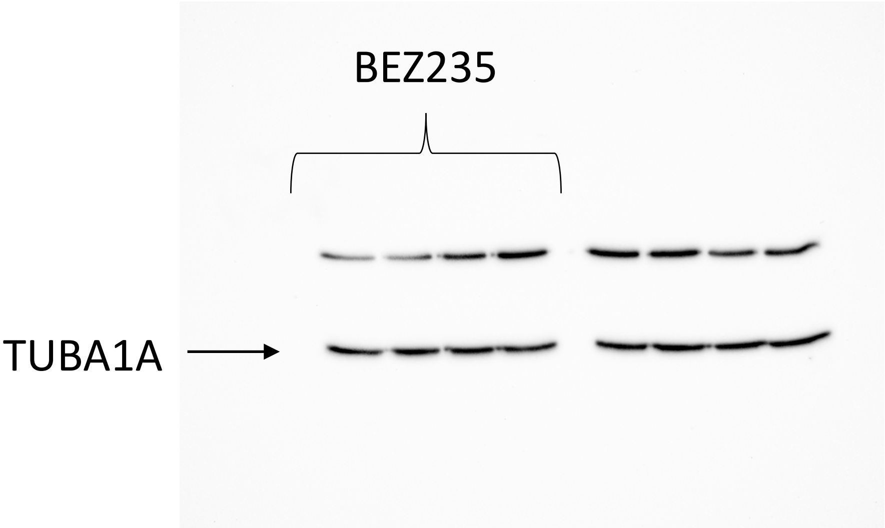

E (DAPT)

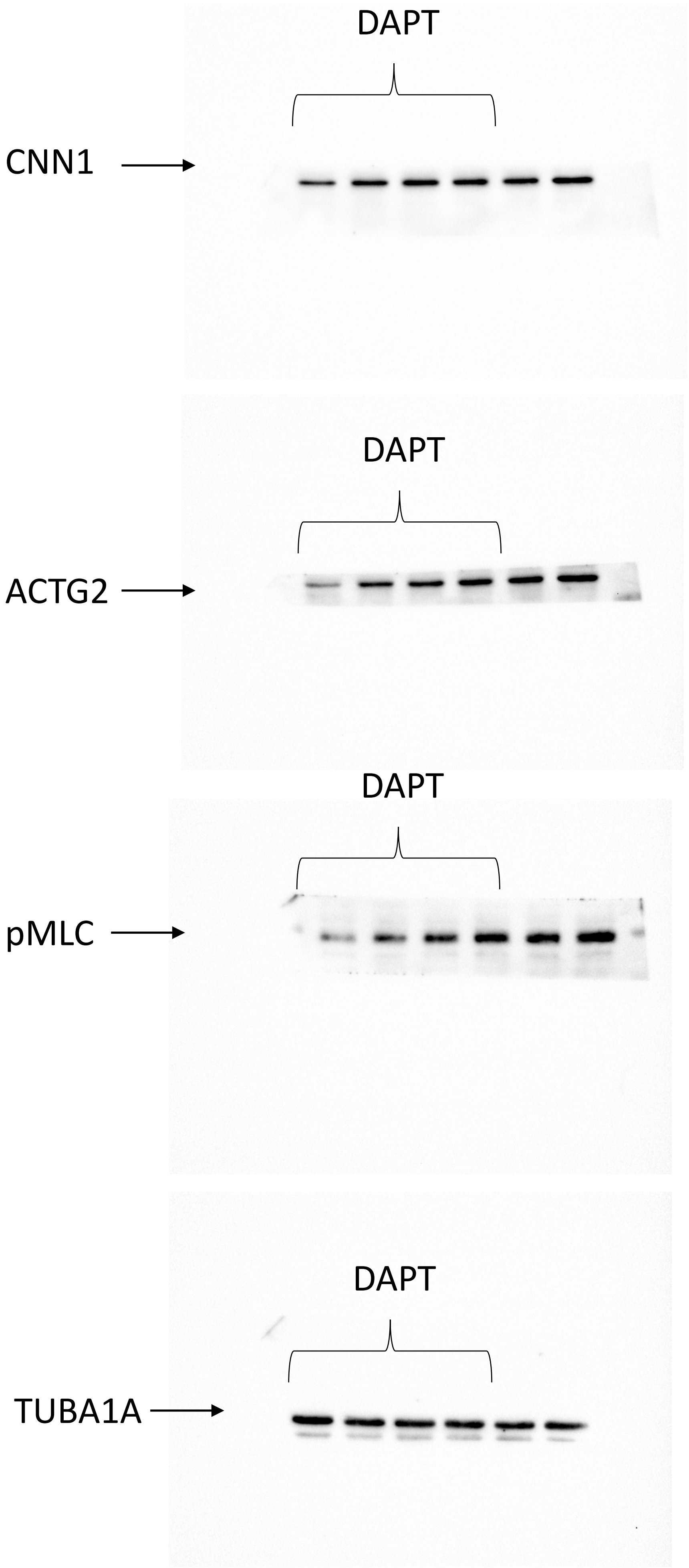

Supplemental Figure 4A

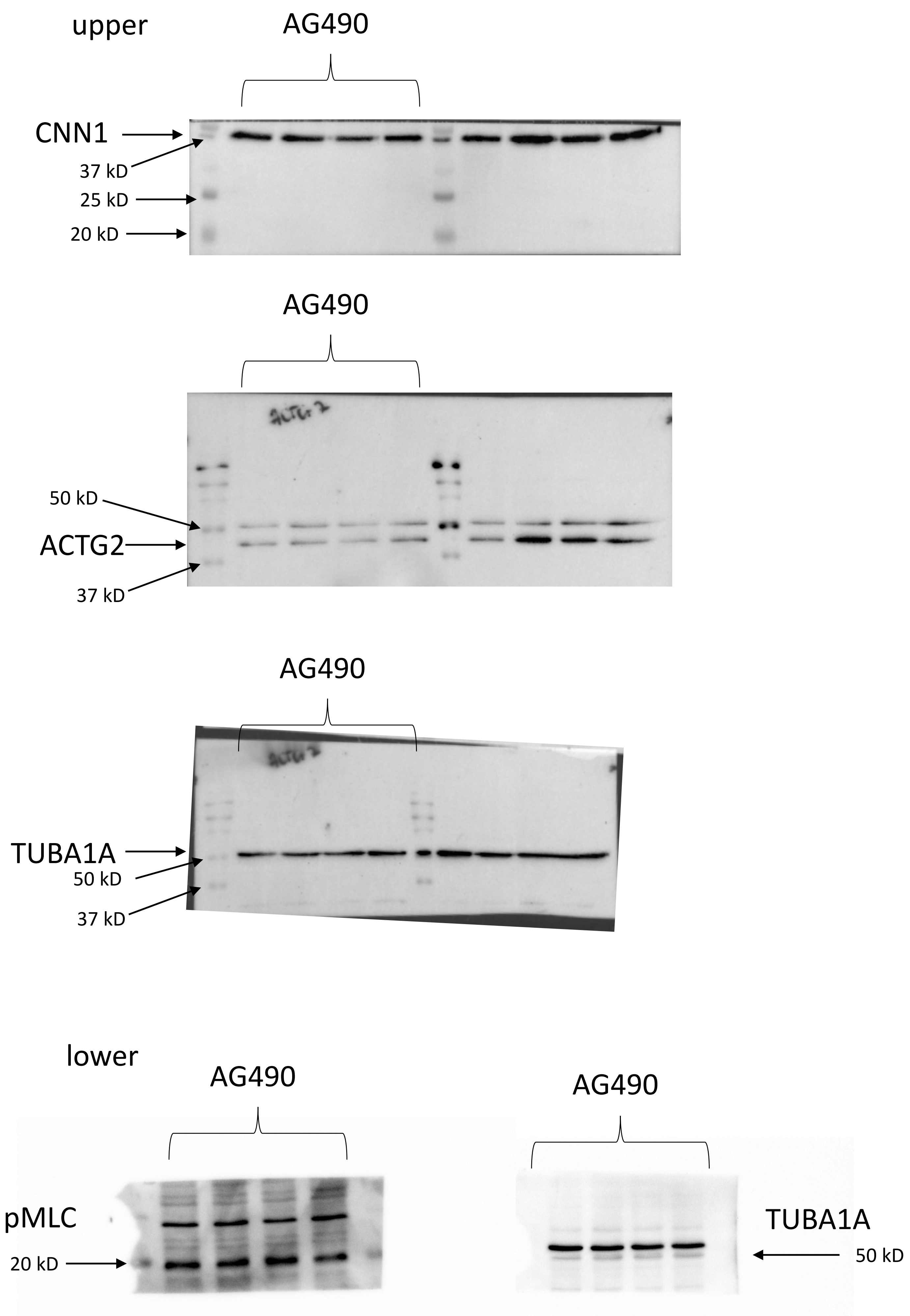

Supplemental Figure 4B

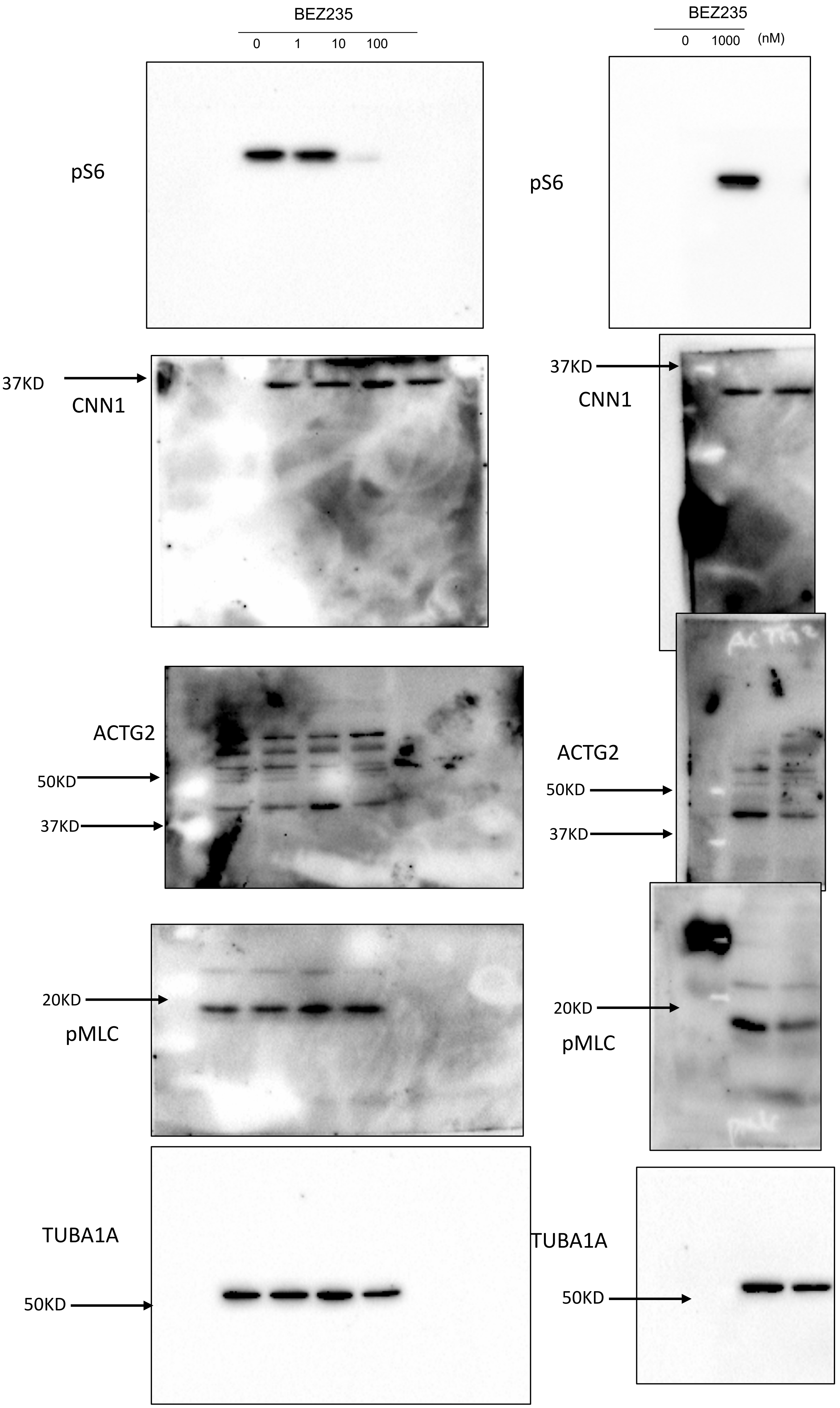

Supplement: Unedited blot and gel images [file jci-134-179860-s237.pdf]
